# The story of clobenpropit and CXCR4: can be an effective drug in cancer and autoimmune diseases?

**DOI:** 10.3389/fphar.2024.1410104

**Published:** 2024-07-12

**Authors:** Mitra Abbasifard, Kowsar Bagherzadeh, Hossein Khorramdelazad

**Affiliations:** ^1^ Department of Internal Medicine, School of Medicine, Ali-Ibn Abi-Talib Hospital, Rafsanjan University of Medical Sciences, Rafsanjan, Iran; ^2^ Eye Research Center, The Five Senses Health Institute, Rassoul Akram Hospital, Iran University of Medical Sciences, Tehran, Iran; ^3^ Department of Immunology, School of Medicine, Rafsanjan University of Medical Sciences, Rafsanjan, Iran

**Keywords:** clobenpropit, CXCR4, autoimmune disease, CXCL12 (SDF-1α), cancer

## Abstract

Clobenpropit is a histamine H3 receptor antagonist and has developed as a potential therapeutic drug due to its ability to inhibit CXCR4, a chemokine receptor involved in autoimmune diseases and cancer pathogenesis. The CXCL12/CXCR4 axis involves several biological phenomena, including cell proliferation, migration, angiogenesis, inflammation, and metastasis. Accordingly, inhibiting CXCR4 can have promising clinical outcomes in patients with malignancy or autoimmune disorders. Based on available knowledge, Clobenpropit can effectively regulate the release of monocyte-derived inflammatory cytokine in autoimmune diseases such as juvenile idiopathic arthritis (JIA), presenting a potential targeted target with possible advantages over current therapeutic approaches. This review summarizes the intricate interplay between Clobenpropit and CXCR4 and the molecular mechanisms underlying their interactions, comprehensively analyzing their impact on immune regulation. Furthermore, we discuss preclinical and clinical investigations highlighting the probable efficacy of Clobenpropit for managing autoimmune diseases and cancer. Through this study, we aim to clarify the immunomodulatory role of Clobenpropit and its advantages and disadvantages as a novel therapeutic opportunity.

## 1 Introduction

The complicated network of molecular communications orchestrating immune responses has long been pivotal in exploring pioneering therapeutic approaches against autoimmune diseases (ADs) and malignancies (Dhillon et al., 2020; Masoumi et al., 2021). Among the numerous players, the chemokine receptor CXCR4 has emerged as a critical mediator in the pathogenesis of various immune-based human disorders (Pozzobon et al., 2016; Bagheri et al., 2019; Kawaguchi et al., 2019). The role of CXCR4 in immune regulation cannot be overstated. CXCR4 involvement in immune cell trafficking (Kucia et al., 2005; Pelekanos et al., 2014), homing (Burger and Bürkle, 2007; Yellowley, 2013; Asri et al., 2016), and cell activation (Kumar et al., 2006; Hong et al., 2009) make it a pivotal player in the orchestration of immune responses (Jacobson and Weiss, 2013). Ligation of CXCL12 to CXCR4 can initiate several downstream signaling pathways, inducing cell growth and proliferation, migration, angiogenesis, inflammation, and metastasis (Hassanshahi et al., 2010; Aminzadeh et al., 2012; Azin et al., 2012; Khorramdelazad et al., 2016; Nazari et al., 2017). Dysregulation of CXCR4 signaling has been associated with ADs, where aberrant immune responses target self-antigens, and tumorigenesis, where uncontrolled cell proliferation and evasion of immune surveillance are hallmarks (Chong and Mohan, 2009; Chatterjee et al., 2014; García-Cuesta et al., 2019; Shi et al., 2020). Therefore, inhibition of the CXCL12/CXCR4 axis can be a potential therapeutic target in cancers and ADs (Chong and Mohan, 2009; Derlin and Hueper, 2018). Numerous studies employed CXCR4 inhibitors to suppress the CXCL12/CXCR4 signals for treating cancer (Domanska et al., 2012; Biasci et al., 2020; Bockorny et al., 2020; Chaudary et al., 2021). AMD3100 is one of the most common CXCR4 inhibitors (De Clercq, 2003). Researchers showed that it could be effective in cancer therapy via the inhibition of tumor cell proliferation and reducing the infiltration of immunosuppressive cells, such as regulatory T cells (Tregs) and myeloid-derived suppressor cells (MDSCs) into the tumor microenvironment (TME) (Scala, 2015; Liu et al., 2021; Lei et al., 2022). Our recent study showed that A1, a novel fluorinated CXCR4 inhibitor, can effectively treat colorectal cancer (CRC) *in vitro* and *in vivo* (Khorramdelazad et al., 2023).

Regarding the potential association between the CXCL12/CXCR4 axis and various signaling pathways, such as the ERK pathway, it has been reported that the CXCL12/CXCR4 signaling pathway plays a proinflammatory role in experimental emporomandibular joint osteoarthritis (TMJOA) model and the bicyclam derivative AMD3100 could reduce the severity of experimental TMJOA (Wang et al., 2016). Current attention has turned towards Clobenpropit, initially recognized as a histamine H3 receptor antagonist, and its exciting proficiency in inhibiting CXCR4 (Mani et al., 2021; Bekaddour et al., 2023). The question here is, in addition to the antihistaminic properties of Clobenpropit, to what extent can this drug help treat CXCL12/CXCR4-related diseases by inhibiting CXCR4? Moreover, what interactions may occur with the simultaneous inhibition of histamine H3 receptor and CXCR4.

This review article aims to summarize the complicated molecular dance between Clobenpropit and CXCR4, clarifying the potential of Clobenpropit as a therapeutic agent in ADs and human malignancies. We will unravel the molecular mechanisms supporting the inhibitory effects of Clobenpropit on CXCR4, exploring its impact on immune cell function and immune-mediated pathologies. Additionally, we will critically analyze preclinical and clinical evidence, evaluating Clobenpropit’s efficacy and safety profile in the most critical ADs and cancer. By doing so, we endeavor to contribute to the developing landscape of targeted therapy, offering new perspectives on utilizing Clobenpropit as a potential therapeutic intervention against diseases characterized by CXCR4-associated immune dysregulation.

## 2 Methodology

### 2.1 Literature search strategy

A comprehensive literature search was conducted across several databases, including PubMed, Scopus, Web of Science, and Google Scholar. The search terms included “Clobenpropit,” “CXCR4,” “histamine H3 receptor antagonist,” “autoimmune diseases,” “cancer,” “CXCL12/CXCR4 axis,” “cell proliferation,” “migration,” “angiogenesis,” “inflammation,” and “metastasis.” The search was limited to articles published in English from 1990 to the present (May 2024).

### 2.2 Inclusion and exclusion criteria

Studies were included in the review if they met the following criteria: Investigated the role of Clobenpropit as a CXCR4 inhibitor; addressed the molecular mechanisms of the CXCL12/CXCR4 axis; examined the effects of Clobenpropit on autoimmune diseases or cancer; included preclinical or clinical data supporting the efficacy of Clobenpropit; peer-reviewed articles, reviews, and clinical trial reports. Moreover, studies were excluded if they did not focus on Clobenpropit or its interaction with CXCR4, were not peer-reviewed, included editorials and commentaries, lacked sufficient methodological detail or experimental rigor, or were unavailable in full-text form.

### 2.3 Data extraction and analysis

Two reviewers independently extracted data using a standardized form. Extracted information included study design, sample size, methods, key findings, and conclusions. Discrepancies between reviewers were resolved through discussion or consultation with a third reviewer.

## 3 The CXCL12/CXCR4 signaling pathway: unraveling developmental and immunological connectivity

As discussed, the CXCL12/CXCR4 signaling pathway is a vital conduit linking developmental processes with immune regulation. Through a complex interplay of chemokine ligand CXCL12 and its receptor CXCR4, this pathway influences diverse cellular behaviors crucial for tissue morphogenesis and immune surveillance (Ara et al., 2005). As we explore the intricate mechanisms and multifaceted roles of the CXCL12/CXCR4 axis in this section, we gain insight into its profound influence on developmental connectivity and immunological responses.

### 3.1 Importance of the CXCL12/CXCR4 axis in development

Hematopoietic stem cells (HSCs) are pivotal in hematopoiesis or generating blood cells (Nemeth and Bodine, 2007). During midgestation, HSCs originate from hemogenic endothelial cells or mesenchymal cells adjacent to the dorsal aorta, migrate to the fetal liver, and colonize the bone marrow (BM) (Nagasawa et al., 1996; Ma et al., 1998; Zou et al., 1998; Ara et al., 2003). Researchers have found that the CXCL12/CXCR4 axis facilitates the BM niche’s hematopoietic stem and progenitor cell (HSPC) colonization during development by genetically modified murine models lacking CXCL12 or CXCR4. In addition, HSC maintenance requires CXCL12/CXCR4 signaling, as evidenced by conditional deletion experiments in BM (Sugiyama et al., 2006; Tzeng et al., 2011; Ding and Morrison, 2013; Greenbaum et al., 2013). Moreover, sperm and oocytes are formed by migrating and colonizing primordial germ cells (PGCs). CXCL12/CXCR4 signaling underlies the colonization of the genital ridges by PGCs, which originate from the allantoic root (Nagasawa, 2014). In murine models, CXCL12/CXCR4 signaling is implicated in colonization (Nagasawa et al., 1996; Zou et al., 1998). Findings in zebrafish further corroborate the significance of CXCL12 in directing PGC migration toward the gonads (Knaut et al., 2003; Nagasawa, 2014).

Signaling between CXCL12 and CXCR4 is fundamental for cardiogenesis and vascular development. Its deficiency impairs membrane formation in the cardioventricular septum and compromises vascularization in multiple tissues (Tachibana et al., 1998; Ara et al., 2005; Li et al., 2013). CXCL12 plays a vital role in patterning vascular pathways during mesenteric development, specifically by facilitating interactions between arterial endothelial cells and the adjacent capillaries (Ara et al., 2005). Zebrafish studies have demonstrated that CXCR4a contributes to forming arterial networks during brain vascularization (Chong et al., 2001; Siekmann et al., 2009). CXCL12/CXCR4 signaling is intricately involved in neurogenesis, influencing migration, differentiation, and axonal guidance. Using mice deficient in CXCL12 or CXCR4, aberrant granule cell clustering in the cerebellum has been observed, hippocampal dentate gyrus morphology has been altered, and the assembly of GABAergic interneurons is disrupted, emphasizing the importance of CXCL12/CXCR4 signaling in neurodevelopmental processes (Ma et al., 1998; Zou et al., 1998; Bagri et al., 2002; Lu et al., 2002; Stumm et al., 2003; Zhu et al., 2009). Additionally, CXCL12 guides the axon trajectory of motor and sensory neurons during the development of the nervous system (Chalasani et al., 2003; Lieberam et al., 2005).

### 3.2 Immunological aspects of the CXCL12/CXCR4 axis

The CXCL12/CXCR4 signaling pathway is critical in immune regulation and exemplifies the nuanced interactions that govern cell migration, homing, and immune surveillance (Lu et al., 2024). This section summarizes the bio-structure, bio-function, and signaling of the CXCL12/CXCR4 axis. At the epicenter of this pathway lies CXCL12, also identified as stromal cell-derived factor-1 α (SDF-1α) (Kukreja, 2005). CXCL12 is a member of the CXC chemokine family, categorized by a conserved cysteine motif that adopts the canonical chemokine fold, comprising three anti-parallel β strands and a single α helix (N terminus post-translational, 2016). In disulfide bonds, the cysteine residues are responsible for the stability of the protein (Xu et al., 2013). The exclusive spatial arrangement of amino acid residues outlines the chemotactic specificity, supporting its ligation with the CXCR4 receptor. The CXCR4 is considered a chemokine receptor for CXCL12 and a seven-transmembrane G protein-coupled receptor (GPCR) (Teixido et al., 2018). CXCR4 possesses a multifaceted structure containing an extracellular N-terminus, seven transmembrane helices, three intracellular loops, and an intracellular C-terminus. The CXCR4 binding pocket contains the N-terminus of CXCL12, starting a cascade of conformational alterations that activate several downstream signaling occurrences (Van Hout, 2019).

**FIGURE 1:**
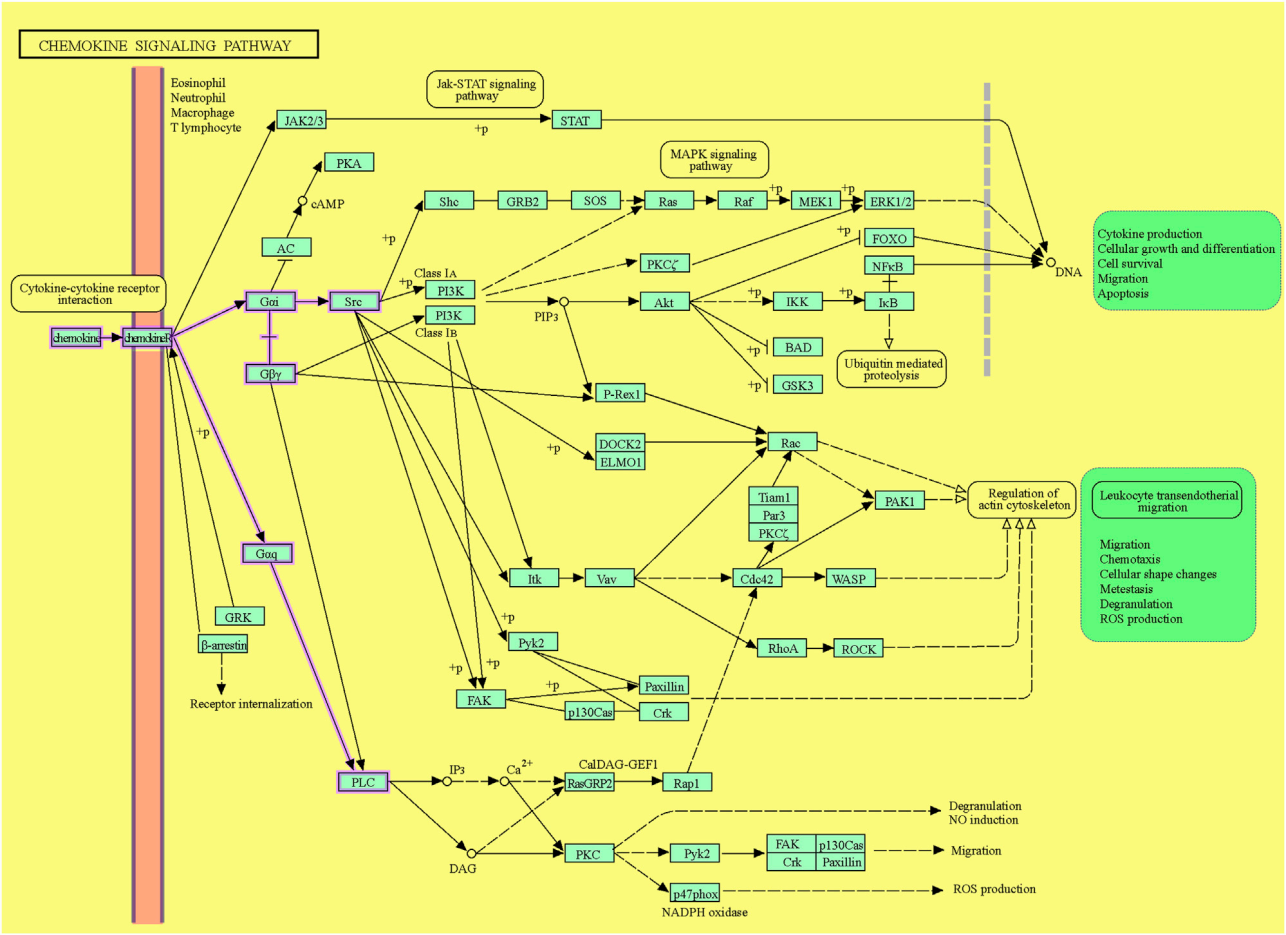
The CXCL12/CXCR4 signaling pathway. The downstream pathways are shown with violet lines (source: KEGG, https://www.kegg.jp/pathway/hsa04062+N01765).

**FIGURE 2:**
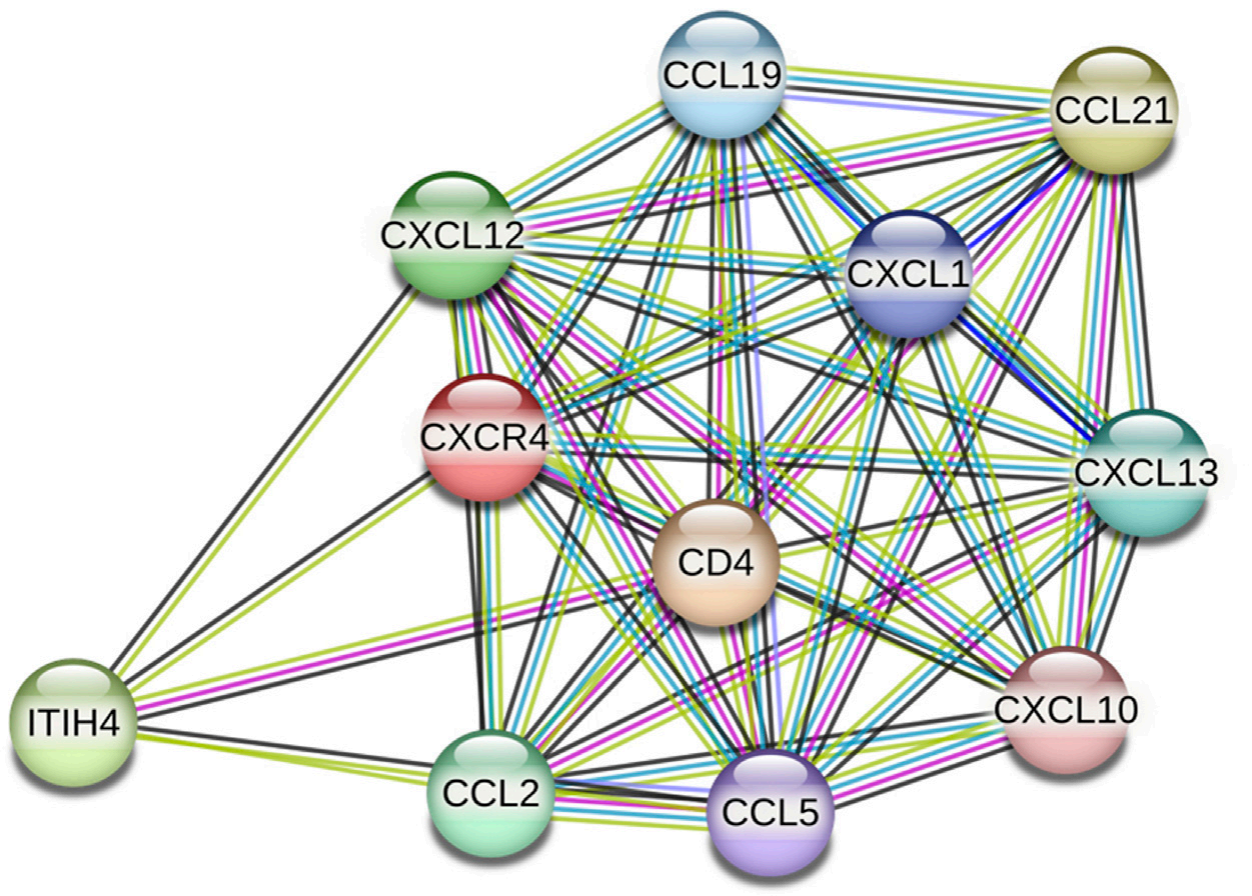
CXCR4 protein interactions (source: STRING, https://stringdb.org/cgi/network?taskId=bT78SZ6aKg1s&sessionId=b9z8Gu1eenbw&allnodes=1).

Following ligation of CXCL12 to CXCR4, a series of bioevents occur, activating downstream signaling pathways that regulate immune cell performance through inducing G-protein pairing and activating heterotrimeric G proteins, principally Gαi (Liekens et al., 2010). Gαi, in turn, hinders adenylyl cyclase, reducing the production of cyclic AMP (cAMP) (Scala, 2015). Condensed cAMP levels modulate intracellular signaling, inducing cell proliferation, differentiation, migration, and survival (Castaldo et al., 2014). Moreover, activating CXCR4 induces the phosphorylation of intracellular adaptor molecules and domains, engaging and triggering kinases such as focal adhesion kinase (FAK) and mitogen-activated protein kinases (MAPK). FAK can regulate cell adhesion and migration, while MAPKs contribute to cell proliferation and survival (Rigiracciolo et al., 2021). The Ras/Raf/MEK/ERK signaling pathway is a prominent downstream branch of MAPK signaling and plays a fundamental role in cellular responses to CXCL12 (Kumari et al., 2021). The phosphoinositide 3-kinase (PI3K) pathway is also employed, activating Akt, which modulates diverse cellular processes, including cell proliferation, metabolism, and survival (West et al., 2002; Mousavi, 2020) (Figure 1). Contributing these signaling cascades in various physiological and pathological states magnificently tunes immune cell responses, pointing out their locomotion, activation, and functions (Mousavi, 2020). As discussed, the CXCL12/CXCR4 axis is crucial in immune cell trafficking, predominantly in hematopoiesis and the homing of immune cells to lymphoid organs and inflammatory milieus (Salcedo and Oppenheim, 2003; Nagasawa, 2014). Evidence revealed that this pathway is critical in embryonic development, organogenesis, and tissue repair (Cheng et al., 2014). Beyond the CXCL12/CXCR4 axis’s physiological functions, its dysregulation concerns several pathological conditions, including cancer metastasis, ADs, and human immunodeficiency virus (HIV) infection (Liekens et al., 2010). The interactions of CXCR4 with other related proteins are illustrated in Figure 2.

Taken together, the CXCL12/CXCR4 axis is a testament to the complicated molecular interactions network that governs immune responses. Therefore, understanding the bio-structure of CXCL12 and CXCR4 offers essential perceptions of their functional roles. The stage-managed activation of downstream signaling pathways orchestrates a symphony of immune responses, emphasizing the pathway’s significance in physiologic and pathologic conditions. As we unravel the intricacies of the immune system and its components, the CXCL12/CXCR4 axis emerges as an essential player, suggesting potential therapeutic targets for several immune-related disorders such as ADs and cancers.

## 4 Role of CXCL12/CXCR4 axis in pathologic states

### 4.1 Cancer

CAFs are believed to be the major producers of CXCL12 in the TME, as they are the most common and significant cells that secrete it (Costa et al., 2014). M2 macrophages and cancer cells also produce CXCL12. By secreting CXCL12, M2 macrophages can activate and differentiate CAF (Liu et al., 2019). Despite being widely expressed by cancer cells from various sources, the CXCR4 receptor is typically found on cancer stem cells (CSCs) and is not present in normal mammary epithelial cells (Yi et al., 2019). This suggests that the CXCL12/CXCR4 axis can function in autocrine and paracrine manners. CXCR4, which promotes embryonic development, is required for CSC migration toward metastatic sites (Huang et al., 2010). Other cells that express CXCR4 include neutrophils, endothelial cells, lymphocytes, stromal fibroblasts, hematopoietic stem cells (HSCs), and MDSCs (Mortezaee, 2020). Accordingly, recruiting CXCR4^+^ MDSCs, M2 macrophages, and Tregs can suppress anti-tumor immune responses, inducing tumor growth and progression.

Genetic mutations and epigenetic changes are at the root of cancer, one of the leading causes of premature death worldwide (Kanwal and Gupta, 2012). It has been revealed that various growth factors and signaling pathways intricately regulate primary tumorigenesis (Cross and Dexter, 1991; Guo et al., 2020). The CXCL12/CXCR4/PI3K/AKT axis is involved in the pathogenesis of several malignancies, such as adamantinomatous craniopharyngiomas, breast cancer (BCa), neuroblastoma, pancreatic intraepithelial neoplasia, medullary thyroid cancer, hepatocellular carcinoma (HCC), colorectal cancer (CRC), and glioblastoma (GB), via activation of different downstream signaling pathways, inducing tumor cell proliferation, migration, and invasion (Carmo et al., 2010; Yin et al., 2019; Yang et al., 2020; Hjazi et al., 2023; Yang et al., 2023). Additionally, CXCR7, a receptor related to CXCR4 and CXCL12, has been associated with the growth and metastasis of tumor cells in colon cancer, melanoma, and BCa (Wang et al., 2015). Metastasis is a major cause of cancer-related mortality that involves sequential invasion, circulation, infiltration, and proliferation (Ha et al., 2013). CXCL12/CXCR4 affects integrin expression, homeobox genes, tight junctions, and matrix metalloproteinases, which affect colorectal, endometrial, breast, and glioma metastasis (Yang et al., 2023).

Several studies have demonstrated that CXCL12 is overexpressed in tumor tissues of various human malignancies (Portella et al., 2021). In addition to fostering pre-metastatic niches (tumorigenic soils), it recruits tumor cells (oncogenic “seeds”) to the niches, inducing tumor progression and metastasis (Yang et al., 2020). Cancer stem/progenitor cells overexpress the CXCR4 receptor, which transmits CXCL12 signals. Oncogenes are activated following the ligation of CXCL12 to CXCR4, which activates multiple downstream pathways. By activating the CXCL12/CXCR4 axis, cancer stem, and progenitor cells are mobilized to pre-metastatic niches and undergo epithelial-mesenchymal transition (EMT) (Yang et al., 2020).

Although it has long been established that cancer-associated fibroblasts (CAFs), the primary producers of CXCL12 in the TME, can induce EMT in tumor cells, the direct involvement of CXCL12 in EMT has remained unclear (Jiang et al., 2023). This ambiguity persists because other factors secreted by CAFs, such as tumor growth factor-beta (TGF-β) and IL-6, are also known to be highly transformative. Interestingly, recent investigations have revealed that overexpression of CXCL12 in MCF7 cell lines (Breast cancer) leads to upregulation of *OCT4*, *Nanog*, and *SOX2* (DiNatale et al., 2022). These factors are well known for their roles in pluripotency and stem cell reprogramming, further confirming the close association between EMT and the stem cell program in cancer. Additionally, CXCL12-driven EMT induction in this model system was found to depend on the Wnt/β-catenin pathway.

Moreover, the significance of CXCL12 in the TME is explored by its interaction with CXCR4, which is often overexpressed in various cancers. This axis stimulates EMT and promotes tumor invasiveness and metastasis (Anastasiadou et al., 2023). The role of the Wnt/β-catenin pathway in this process highlights a critical signaling mechanism that integrates external signals from the TME with intracellular pathways governing cell differentiation and proliferation (Moon, 2005). Studies have reported that activating the Wnt/β-catenin pathway can stabilize β-catenin in the cytoplasm, translocating to the nucleus, and the subsequent activation of target genes that promote EMT and stemness properties (Jiang et al., 2007). In addition to CXCL12, the interplay between CAFs and tumor cells involves a complex network of signaling molecules. For instance, TGF-β has been extensively studied for its dual role in cancer. It acts as a tumor suppressor in the early stages and a metastasis promoter in the advanced stages (Pardali and Moustakas, 2007). Similarly, IL-6 is known to activate the JAK/STAT3 signaling pathway, contributing to EMT and cancer progression (Pardali and Moustakas, 2007). Understanding the specific contributions of these factors, including CXCL12, within the TME is crucial for developing targeted therapies to disrupt these pro-tumorigenic interactions.

Collectively, the evidence suggests that CXCL12 plays a significant role in inducing EMT and stemness in tumor cells, with the Wnt/β-catenin pathway being a crucial mediator in this process. Further studies are required to fully elucidate how CXCL12 and other CAF-derived factors contribute to tumor progression and explore potential therapeutic strategies targeting these pathways (Shan et al., 2015).

**FIGURE 3:**
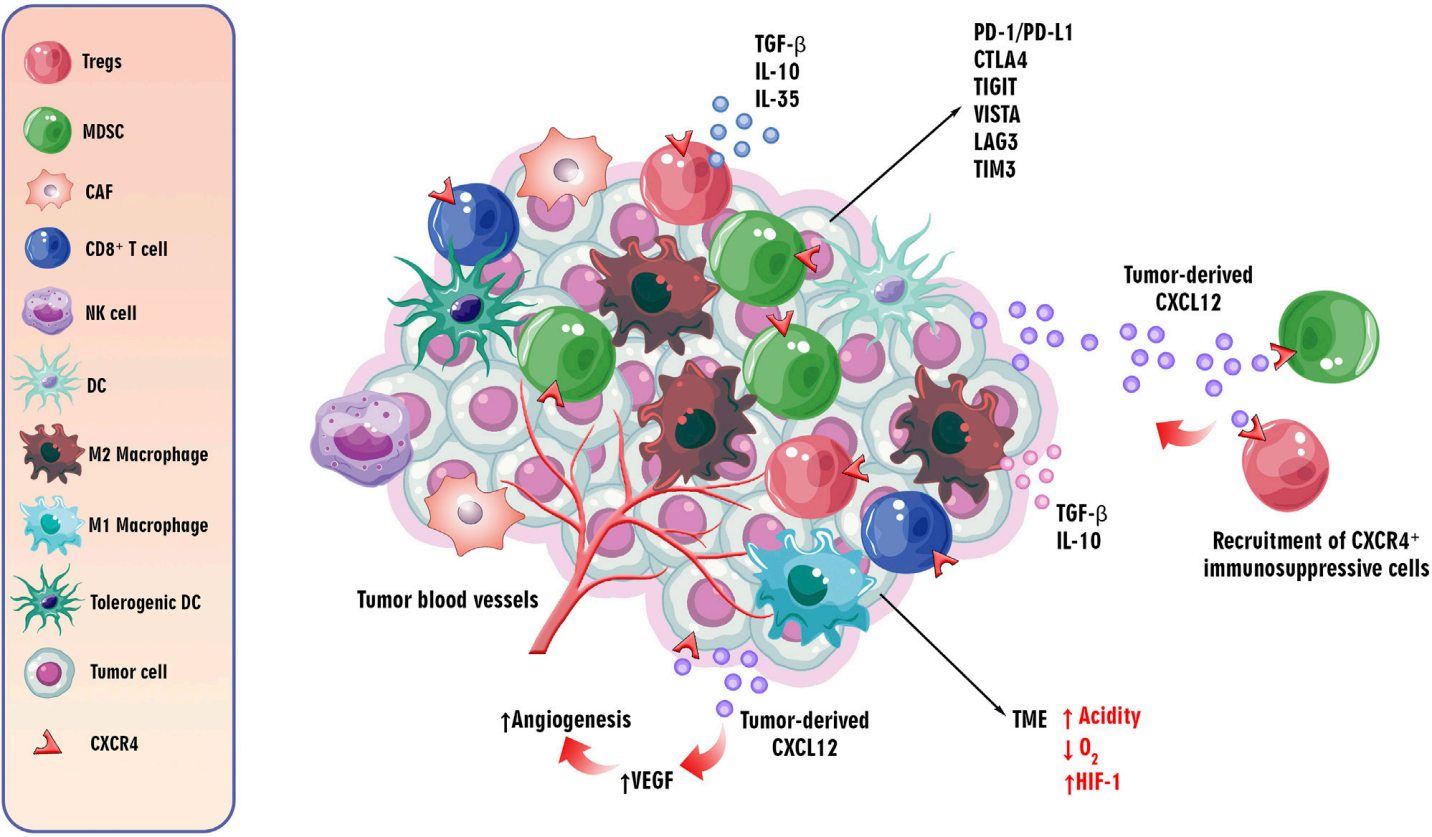
The role of/CXCL12CXCR4 axis in the TME. The critical features of the TME influence cancer progression. The TME is characterized by hypoxia, high acidity, and expression of inhibitory immune checkpoints such as PD-1/PD-L1, CTLA-4, LAG-3, TIM-3, TIGIT, and VISTA. Additionally, immunosuppressive cells like Tregs, TAMs, CAFs, and MDSCs are recruited, suppressing anti-tumor immune responses via secreting immunosuppressive cytokines, such as IL-10, IL-35, and TGF-β. Chemokines, particularly the dysregulated CXCL12/CXCR4 axis, play a role in recruiting regulatory and cancer-associated cells to the TME. The CXCR4 receptor, upregulated in hypoxic conditions, induces angiogenesis through the PI3K/AKT pathway, contributing to tumor progression by promoting VEGF expression. This axis is implicated in both tumor angiogenesis and metastasis to distant organs.

According to the available knowledge, the TME plays a pivotal role in cancer progression. The hypoxic condition and high acidity, as well as expressing inhibitory immune checkpoints, such as programmed cell death protein 1/programmed cell death ligand 1 (PD-1/PD-L1), anti-cytotoxic T lymphocyte-associated antigen-4 (CTLA-4), like lymphocyte activation gene-3 (LAG-3), T cell immunoglobulin and mucin-domain containing-3 (TIM-3), T cell immunoglobulin and ITIM domain (TIGIT), and V-domain Ig suppressor of T cell activation (VISTA) are the essential features of the TME (Qin et al., 2019). Moreover, recruitment and infiltration of immunosuppressive immune cells, such as regulatory T cells (Tregs), tumor-associated macrophages (TAMs), cancer-associated fibroblasts (CAFs), and myeloid-derived suppressor cells (MDSCs) in the TME can suppress anti-tumor immune responses to tumor cells via releasing immunosuppressive mediators, such as IL-10, IL-35, and tumor growth factor beta (TGF-β) (Gao et al., 2023) (Figure 3). Chemokines recruit anti and pro-tumor immune and non-immune cells into the TME. Dysregulation of CXCL12 secretion by tumor cells and expression of CXCR4 by immunosuppressive cells induces the creation of an inhibitory TME by fostering the infiltration of the mentioned regulatory and cancer-associated cells (Mortezaee, 2020).

As discussed, the CXCL12/CXCR4 axis is involved in tumor angiogenesis and metastasis of tumor cells to distant organs. By inducing vascular endothelial growth factor (VEGF) through the PI3K/AKT pathway, the CXCR4 receptor can induce tumor angiogenesis, an essential step in tumor progression (Ghalehbandi et al., 2023). Furthermore, the upregulation of CXCR4 in hypoxic conditions and its role in hypoxia-inducible factor-1 α (HIF-1 α)-induced VEGF expression can lead to angiogenesis in the TME (Kruszyna et al., 2022) (Figure 3). Accordingly, understanding the complicated mechanisms underlying tumorigenesis, progression, and the role of the CXCL12/CXCR4 axis in these processes is essential for emerging targeted therapeutic interventions in cancer therapy.

### 4.2 Autoimmune diseases

**FIGURE 4:**
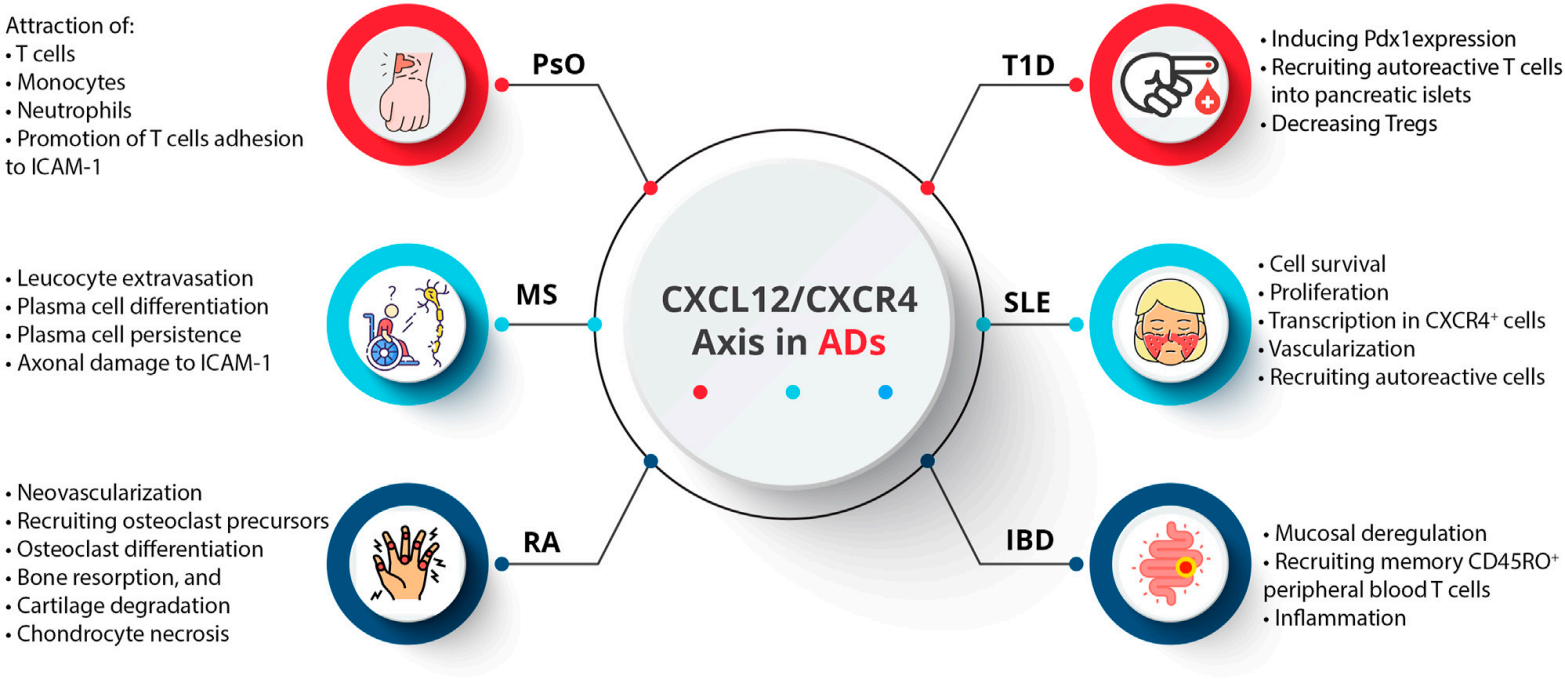
Dysregulation of the CXCL12/CXCR4 axis in ADs.

An AD occurs when the immune system components, such as autoantibodies and autoreactive T cells, incorrectly target specific self-antigens, damaging tissues and organs (Muñoz-Carrillo et al., 2018a). These diseases are not generalized attacks but are mediated by immune responses directed against specific body parts (Muñoz-Carrillo et al., 2018b). A particular AD targets a particular tissue or organ, resulting in diverse clinical symptoms (Pisetsky, 2023). In addition, a majority of ADs appear to have a relapsing/remitting course, where periods of active disease (flare-ups) alternate with periods of remission. Symptoms worsen during flare-ups due to increased immune activity, while symptoms decrease during remissions due to reduced immune activity (Lebel et al., 2023). As a result of genetic predisposition, environmental triggers (smoking, chemical compounds, infectious agents, radiation, and ultraviolet light), and dysregulation of immune tolerance, the underlying pathophysiological mechanisms often fail to differentiate self from non-self (Javierre et al., 2011; Capalbo et al., 2012). Therefore, diagnosing and managing these diseases requires understanding their specific target antigens and dynamic nature (Muñoz-Carrillo et al., 2018a). Activated innate immune cells, stromal cells, and tissue cells produce cytokines and chemokines, which regulate immune cell trafficking and play a significant role in ADs (Shachar and Karin, 2013; Elemam et al., 2020; Fallahi et al., 2020; Abassifard et al., 2021; Moadab et al., 2021; Abbasifard et al., 2023). Although CXCL12 was initially regarded as a homeostatic chemokine, it also plays a vital role in inflammation. Inflammatory bowel disease (IBD), multiple sclerosis (MS), rheumatoid arthritis (RA), psoriasis (PsO), type 1 diabetes (T1D), and systemic lupus erythematosus (SLE) are among the ADs that are implicated by the CXCL12/CXCR4 axis (García-Cuesta et al., 2019) (Figure 4).

CXCL12 plays a crucial role in skin homeostasis and inflammation (Abdelaal et al., 2020). In PsO as a chronic inflammatory skin disease, inflammatory leukocytes, including dendritic cells (DCs), macrophages, and T cells, accumulate, leading to pronounced inflammatory angiogenesis (Gulletta et al., 2013). VEGF-A upregulates the expression of CXCL12 in psoriatic lesions. Psoriatic skin lesions also exhibit elevated mRNA levels of *CXCR4* and *CXCL12* (Petit et al., 2007; Suárez-Fariñas et al., 2011). A study evaluated CXCL12 expression in PsO vulgaris and psoriatic arthritis (PsA) patients concerning disease activity and methotrexate (MTX) therapy, and findings showed significantly higher CXCL12 expression in PsA patients compared to PsO vulgaris patients before treatment but not after. Post-MTX therapy, PsO vulgaris patients showed a significant decrease in CXCL12 expression, while PsA patients did not show a significant change. The reduction in PASI scores correlated moderately with decreased CXCL12 expression in PsO patients. Accordingly, CXCL12 may be involved in the progression from PsO vulgaris to PsA, with MTX therapy reducing CXCL12 expression and disease severity, suggesting CXCL12 as a potential biomarker for psoriasis severity (Abdelaal et al., 2020).

MS is a demyelinating disease characterized by inflammation, progressive myelin loss within the central nervous system (CNS), and failure to remyelinate damaged axons (Carbajal et al., 2010). Leukocytes need to penetrate the brain parenchyma for tissue injury, and the unique CNS barriers challenge immune cell activation (Perry et al., 1997). CXCL12, constitutively expressed in the adult CNS and upregulated under pathological conditions, orchestrates leukocyte trafficking in the CNS (Durrant et al., 2014). Chemokines, receptors, and adhesion molecules orchestrate leukocyte trafficking (Olson and Ley, 2002). In MS patients, CXCL12 levels are elevated in serum and cerebrospinal fluid and expressed in active lesions, suggesting its involvement in disease pathology (Azin et al., 2012; Khorramdelazad et al., 2016; Bagheri et al., 2019; Marastoni et al., 2021). It has been revealed that CXCL12 localization on blood vessels specifies a potential role in leucocyte extravasation, and the CXCL12/CXCR4 axis may contribute to plasma cell differentiation and persistence. In addition, following the cleavage of CXCL12 by metalloproteases, it can convert to a neurotoxic mediator that can damage axons (Krumbholz et al., 2006).

Issues in MS suggest deficiencies in recruiting and maturing oligodendrocyte progenitor cells (OPCs), indicating the crucial role of cell replacement therapies in improving remyelination (Hughes and Stockton, 2021). In a study using a model of viral-induced demyelination, the signaling cues guiding the migration of transplanted remyelination-competent cells were investigated (Carbajal et al., 2010). While rodent-derived glial cell transplantation in MS models has been successful, the mechanisms of cell navigation within the inflammatory environment created by persistent viruses are poorly understood. The JHM strain of mouse hepatitis virus (JHMV) infection in mice induced an immune-mediated demyelinating disease similar to MS. Surgical engraftment of GFP^+^ neural stem cells (NSCs) into the spinal cords of JHMV-infected mice resulted in migration, proliferation, and differentiation into OPCs and mature oligodendrocytes, inducing axonal remyelination. Using anti-CXCL12 blocking serum significantly reduced the migration and proliferation of engrafted stem cells. Additionally, CXCR4 antagonists, but not CXCR7, similarly inhibited migration and proliferation (Carbajal et al., 2010). These outcomes emphasize the pivotal role of the CXCL12/CXCR4 axis in recruiting engrafted stem cells to damaged CNS sites in mice with immune-mediated demyelination due to persistent viral infection.

RA is an inflammatory autoimmune disease that primarily affects the joints. This type of AD involves synovial fibroblasts, endothelial cells, and chronic inflammation (Masoumi et al., 2023). The pathogenesis of RA involves a complex interplay between immune cells and cytokines (Kondo et al., 2021). It has been revealed that activated macrophages and synovial fibroblasts are activated by T cells, causing the release of pro-inflammatory cytokines like TNF-α and IL-17 (Tu et al., 2022). Releasing pro-inflammatory cytokines and chemokines by activated macrophages contributes to inflammation and joint damage (Moadab et al., 2021). A crucial aspect of RA is the degradation of bone and cartilage, which occurs due to synovial fibroblasts and macrophages secreting matrix metalloproteinases (MMPs) (Lefevre et al., 2015). As a result, cartilage and the bones beneath are destroyed by MMPs that destroy extracellular matrix components. T cells and macrophages collaborate on this coordinated attack, facilitated by MMPs, highlighting the importance of targeting these pathways in therapeutic interventions against RA (Siouti and Andreakos, 2019). It has been shown that chronic inflammation and bone erosion are related to the CXCL12/CXCR4 axis, contributing to bone and cartilage damage (Peng et al., 2020). It is associated with CXCL12 that neovascularization occurs in inflamed RA joints, particularly in their early stages (Yu et al., 2003). As a result, immune cells in the synovium express CXCR4. CXCL12 also induces recruiting osteoclast precursors, stimulating differentiation, bone resorption, and cartilage degradation (Grassi et al., 2004; Wright et al., 2005). Hypoxia stimulates VEGF and CXCR4 expression in inflamed joints by activating HIF-1 (Imtiyaz and Simon, 2010). In addition, *in vitro* experiments revealed that CXCL12 enhances chondrocyte necrosis, signifying the role of this CXC chemokine in cartilage damage (Xu et al., 2012).

MicroRNAs (miRs) play a significant role in the initiation and progression of RA, though the specific functions and mechanisms of miR-23 in RA are not fully understood (Evangelatos et al., 2019). An investigation demonstrated that miR-23 was downregulated, while CXCL12 was upregulated in RA samples compared to control samples (Gao et al., 2021). Overexpression of miR-23 suppressed inflammation by reducing TNF-α, IL-1β, and IL-8 expression. Mechanistically, miR-23 decreased *CXCL12* mRNA expression by binding to its 3′-untranslated region, and overexpression of CXCL12 counteracted the anti-inflammatory effects of miR-23 mimic. Additionally, CXCL12 promotes inflammation by activating NF-κB signaling (Gao et al., 2021). Therefore, miR-23 alleviates RA inflammation by regulating CXCL12 via the NF-κB pathway, suggesting that targeting miR-23 could be a potential strategy for diagnosing and treating RA.

Another investigation found that the levels of CXCR4 and CXCL12 in the serum and joint synovial fluid were significantly higher in patients with RA than in normal subjects. These levels were also higher in the RA-active group compared to both the remission and control groups. A positive correlation was also observed between the expression of CXCR4 and CXCL12 and the erythrocyte sedimentation rate (ESR), C-reactive protein (CRP), rheumatoid factor (RF), and Disease activity score in 28 joints (DAS28) scores. These outcomes suggest that CXCR4 and CXCL12 are highly expressed in RA patients, with their levels correlating positively with these clinical markers of disease activity (Peng et al., 2020).

SLE is characterized by immune complexes of autoantibodies and autoantigens circulating in the blood, leading to an inflammatory process and organ damage (Abbasifard et al., 2020). It has been revealed that chemokines, including CXCL9, CXCL10, CXCL12, and CXCL13, play crucial roles in the pathogenesis of SLE (Pan et al., 2022). The expression of CXCR4 is upregulated in various immune cell types, such as monocytes, neutrophils, T cells, B cells, and plasma cells (Badr et al., 2015). Moreover, CXCL12 expression is elevated in the kidney. CXCR4 is upregulated in SLE patients, suggesting it may be a therapeutic target for SLE patients with kidney and CNS involvement (Wang et al., 2010; Badr et al., 2015). In contrast, circulating B cells from SLE patients show altered migration and distribution of B cell compartments due to the downregulation of CXCR4 (Biajoux et al., 2012). The signaling cascades involving PI3K/AKT, MAPKs (ERK, JNK, p38), and the regulation of NF-κB nuclear translocation (IκBs) are critically involved in B cell differentiation and the production of autoantibodies during SLE disease progression (Sen et al., 2014).

However, it appears that inhibiting the CXCR4/CXCL12 axis could mitigate the autoimmune response and inflammation associated with SLE. Studies in lupus-prone murine models demonstrated that CXCR4 was upregulated in B cells, monocytes, neutrophils, and plasma cells, driven by toll-like receptors (TLRs) and pro-inflammatory cytokines. By upregulating this pathway, B cells were able to survive and migrate towards gradients of CXCL12 (Balabanian et al., 2003; von Hofsten et al., 2024).

A previous study found that NZB/W mice susceptible to lupus had elevated CXCL12 levels in the kidneys, contributing to lupus nephritis (Balabanian et al., 2003). Similar findings in other mouse models (B6.Sle1.Yaa, BXSB, MRL.lpr) were also confirmed (Wang et al., 2009). However, these findings are not replicated in human SLE patients (Wang et al., 2009). Studies report that B cells and CD4^+^ T cells express high levels of CXCR4 when disease severity is high, while others conclude that low levels of CXCR4 in specific lymphocyte subsets are associated with disease severity (Chong and Mohan, 2009). Discrepancies may be attributed to sample size differences and patient characteristics. Rather than measuring CXCR4 and CXCL12 levels in peripheral blood, end organs could provide more insights into their role in SLE. Biopsies of lupus nephritis and cutaneous lupus skin demonstrate increased CXCL12 levels correlated with disease severity. The migration of CXCR4^+^ cells into these organs may explain why some studies report lower levels of CXCR4 in peripheral blood (Chong and Mohan, 2009).

SLE-associated glomerulonephritis is accompanied by hyperplastic kidney lesions caused by CXCR4 dysregulation in kidney epithelial cells (Rizzo et al., 2013). This interaction may be crucial during lupus in attracting these cells to the kidney and skin, which are affected peripherally. In addition, CXCL12 binding to CXCR4 boosts cell survival, proliferation, and transcription, based on studies with mice lacking either CXCL12 or CXCR4. In mice with defective CXCL12 or CXCR4, vascularization, bone marrow myelopoiesis, and limb innervation have been observed. As a result of these findings, CXCL12/CXCR4 interactions play a crucial role in numerous physiological processes and are likely to significantly impact pathological conditions such as lupus (Wang et al., 2010).

Several chemokines are associated with T1D, including CXCL10, CCL5, CCL8, CXCL9, and CX3CL1, which are involved in insulin metabolism and pancreatic β-cell destruction (Overbergh et al., 2006). CXCL12/CXCR4 signaling is critical Field (Oliver-Krasinski et al., 2009) for the pancreatic islet to develop and differentiate. CXCL12 induces pancreatic and duodenal homeobox 1 (Pdx1) expression in the pre-pancreas region by attracting CXCR4-expressing angioblasts. All islet cell types must be formed to express neurogenin 3 (Ngn3) via Pdx1 (Oliver-Krasinski et al., 2009). CXCL12 is also vital in influencing immune processes and directing T-cell migration. Recruiting autoreactive T cells into pancreatic islets causes insulitis and T1D. According to most studies, CXCL12 inhibition inhibits diabetes progression and insulitis, but conflicting reports suggest that adoptive cell transfer may protect against diabetes. Among the unique properties of CXCL12 are that it induces bidirectional movement of T cells and exerts a chemorepulsive effect on diabetogenic T cells while promoting normal T cell adhesion (Vidaković et al., 2015). By expressing CXCL12 in islets, autoreactive T cells are selectively repelled, and Treg cells are retained at the site. Tregs are critical in suppressing autoimmunity and are implicated in the development of T1D. It has been demonstrated that the absence of Tregs in pancreatic lymph nodes (PLNs) is correlated with T1D in non-obese diabetic mice. The restoration of euglycemia is associated with the recovery of Treg populations in PLNs, which is linked to a decrease in CXCL12 expression. In order to treat T1D effectively, we may need to enhance the CXCL12/CXCR4 axis and retain Tregs in PLNs (Vidaković et al., 2015).

Recently, an investigation reported that CD57^+^ CD8^+^ T cells, also known as effector memory cells, contribute to tumor and virus immunity and are associated with autoimmunity (Zhong et al., 2024). However, there needs to be more knowledge of how they contribute to T1D. Upon examining a T1D patient with a STAT3 mutation, these cells were observed to be increased. The CD57^+^ CD8^+^ T cells of T1D patients undergo significant changes during disease progression. In longitudinal studies, their prevalence was associated with declining function of the CD4^+^ cells. There is evidence that these cells are critical to the pathophysiology of T1D, as they produce cytotoxic cytokines, increase glucose uptake, and produce pro-inflammatory cytokines. Erk1/2 signaling enhances CD57^+^ CD8^+^ T cell expansion and function *in vitro* via the CXCL12/CXCR4 axis. Changes in serum CXCL12 levels were noted during the peri-remission phase of T1D. T1D mice treated with LY2510924, a CXCR4 antagonist, showed reduced infiltration of T cells and improved insulin sensitivity (Zhong et al., 2024). Based on these findings, CD57^+^CD8^+^ T cells play a crucial role in driving T1D responses, which may be a potential therapeutic option to delay the progression of the disease.

An essential function of intestinal epithelial cells (IECs) in the normal intestinal mucosa is migration, barrier maturation, and restitution, which are all mediated by cAMP. In recent studies, CXCR4 and CXCL12 are present in lamina propria T cells (LPTs), which have been implicated in IBD pathogenesis. IECs of IBD patients express CXCR4 more, and CXCL12 is upregulated in inflamed mucosa. In all sources of peripheral blood T cells (PBTs) and LPTs, CXCL12 functions as a potent chemoattractant, whether they are normal or IBD-related. As evidenced by the accumulation of CXCR4^+^ cells near CXCR12-expressing IECs, interactions between CXCL12 and CXCR4 contribute to mucosal deregulation, specifically impacting memory CD45RO^+^ LPTs (Werner et al., 2013). CXCL12 directs the proliferation of epithelial endocrine precursor cells in the human development of islet cells and phosphatidyl inositol-3 and AKT kinase (Oliver-Krasinski et al., 2009; Weir et al., 2011). It has been found that some IBDs, such as ulcerative colitis and Crohn’s disease, are caused by dysregulated immune responses in a genetically susceptible individual in response to environmental triggers (Scharl and Rogler, 2012; Wallace et al., 2014). It has been shown that intestinal epithelial cells and lamina propria cells express CXCL12 and CXCR4 upregulation in IBD patients. The CXCL12/CXCR4 axis is associated with IBD progression and severity, as it recruits memory Th1 cells, particularly T cells (Agace et al., 2000; Katsuta et al., 2000). It has been reported that polymorphisms in this axis contribute to the recruitment of memory Th1 cells (Mrowicki et al., 2014).

Collectively, it should be noted that the dual role of the CXCL12/CXCR4 axis in ADs like SLE and T1D indicates that targeting this axis may not always be practical. Increased CXCL12 levels in lupus-prone mice have been associated with improved SLE symptoms when treated with specific peptides, suggesting its role in the disease’s pathogenesis (Chong and Mohan, 2009). In T1D, the CXCL12/CXCR4 axis plays a significant role in promoting pancreatic β-cell survival. Studies show that CXCL12 helps protect β-cells from apoptosis and streptozotocin (STZ)-induced diabetes by activating the AKT pathway, which promotes cell survival. Blocking CXCR4 induces apoptosis and reduces cell survival markers in β-cells, while overexpressing CXCL12 in β-cells enhances resistance to apoptosis and diabetes (Yano et al., 2007). However, the phenotype of recruited CXCR4^+^ cells can be crucial in the ADs pathogenesis. These findings highlight the complexity of the CXCL12/CXCR4 axis, which can contribute to disease pathogenesis in SLE and offer therapeutic benefits in T1D, suggesting that blanket targeting of this axis may not be universally beneficial.

## 5 Targeting CXCR4 in pathologic conditions

This section summarized the importance of receptor and ligand inhibition in all types of diseases and autoimmune diseases.

### 5.1 Cancer

The CXCL12/CXCR4 axis plays a pivotal role in tumor progression and metastasis, making it a promising therapeutic target in cancer. By interacting with its receptor CXCR4, CXCL12 promotes migration, invasion, and angiogenesis in cancer cells, a chemokine abundantly expressed in the TME. This ligation is disrupted by inhibitors, which inhibit metastatic spread and stimulate antitumor immune responses. Various cancers have shown promising outcomes when CXCR4 inhibitors are used, such as AMD3100 (Zhou et al., 2020). Thus, the CXCL12/CXCR4 axis has become a compelling target for novel cancer therapies to enhance treatment efficacy and prevent metastasis.

Considering the role CXCR4 plays in tumor progression and metastasis, its inhibition offers significant potential for cancer treatment (Chatterjee et al., 2014). The expression of CXCR4 is associated with increased invasiveness and distant metastases in several cancers (Yang et al., 2020). It has been shown in preclinical and early clinical studies that blocking CXCR4 can disrupt the interaction between cancer cells and the microenvironment, preventing cancer cells from migrating to secondary sites (Zlotnik, 2008). A strategy to impede cancer progression and improve treatment outcomes is effective in early clinical studies targeting CXCR4. Due to these findings, CXCR4 inhibitors are being explored as potential cancer therapies that could impede metastasis and boost cancer therapy efficacy.

An investigation of patients with microsatellite stable pancreatic (PDA) or CRC who failed to respond to immune therapy using T cell checkpoint inhibitors in the context of cancer biology (Biasci et al., 2020). Based on their observations that cancer cells in these tumors are coated with CXCL12, the researchers proposed a possible explanation for this lack of response. Further, they note that stimulation of CXCR4 inhibited the migration of these immune cells mediated by other chemokines. For 7 days, the researchers continuously infused AMD3100 to patients to assess the relevance of these findings. Using transcriptomic analysis, they compared biopsies taken before and after treatment of metastatic lesions. This immunological response, which appears to be predictive of a positive clinical response to T-cell checkpoint inhibition, was found to be induced by the CXCR4 inhibitor. The non-response to immunotherapy may be linked to CXCL12 in cancer cells in some pancreatic and CRCs with specific characteristics (microsatellite stable). Researchers observed that AMD3100 inhibited the tumor’s immune response, making it more susceptible to T cell checkpoint inhibitors by disrupting its immune response (Biasci et al., 2020). These findings suggest that targeting the CXCR4 pathway could enhance immunotherapy effectiveness in these cancer types. As a result of intrinsic/acquired resistance, antiangiogenic therapies provided limited survival benefits to cancer patients (Haibe et al., 2020). It was crucial for improving treatment outcomes to understand and target resistance mechanisms, especially in cancers that required antiangiogenic therapy, such as colon cancer. Anti-VEGFR2 treatment increased CXCL12/CXCR4 expression in orthotopic CRC models and conditional *Apc* mutant spontaneous rectal tumors (Jung et al., 2017). In response to CXCR4 signaling, anti-VEGFR2 innate immune cells were recruited to the CRCs, including Ly6C^low^ monocytes and Ly6G^+^ neutrophils (Jung et al., 2017). These pathways can also be successfully targeted genetically and pharmacologically, including AMD3100, which significantly enhanced response to treatment. These strategies can be readily translated into the clinic. The effectiveness of PD-1 inhibitors in pancreatic ductal adenocarcinoma (PDAC) is limited, suggesting alternative pathways should be explored (Kabacaoglu et al., 2018).

In metastatic PDAC, BL-8040 was combined with pembrolizumab and chemotherapy in a study (Bockorny et al., 2020). There was a 34.5% disease control rate (DCR) in cohort 1 (chemotherapy-resistant patients), as well as a median overall survival (mOS) of 3.3 months in second-line therapy. As a result of BL-8040, CD8^+^ T cells were infiltrated in cohort 2 more efficiently, and immunosuppressive cells were reduced. Combined with pembrolizumab and chemotherapy, the tumor objective response rate (ORR) was 32%, DCR was 77%, and the median response duration was 7.8 months. A randomized trial is needed to confirm the effectiveness of dual CXCR4 and PD-1 blockade in PDAC (Bockorny et al., 2020). An investigation was conducted to assess the efficacy of a combined approach incorporating radiation therapy (RT) with cisplatin (RTCT) and the CXCR4 inhibitor X4-136, which was considered suitable for clinical use (Chaudary et al., 2021). This study found that RTCT alone increased CXCL12/CXCR4 signaling, intratumoral accumulation of myeloid cells, and PD-L1 expression.

In contrast, X4-136 was introduced along with RTCT to counteract these effects, enhancing the primary tumor response and reducing metastases. Furthermore, X4-136 alleviated late histologic changes caused by delayed RT toxicity by reducing acute toxicity in intestinal crypt cells. Study findings indicate that this combination therapy can minimize adverse effects on normal tissues, including the intestines, while improving cervical cancer treatment outcomes. It is suggested that clinical trials should be conducted to explore these benefits further, as well as the possibility of applying this approach to other cancer types for which RTCT is a curative (Chaudary et al., 2021).

It has been shown that immune checkpoint blockade (ICB) therapies are less effective in triple-negative breast cancer (TNBC) due to insufficient T cell infiltration. Immunostimulatory approaches have been developed in the field (Kruszyna et al., 2022) to enhance ICB response. As part of a novel strategy aimed at improving AMD3100’s therapeutic efficacy (Lu et al., 2021), a liposomal formulation targeting CXCR4 was developed. A dual blocker and targeting moiety, AMD3100 acted both extracellularly and intracellularly to inhibit CXCR4 activation. AMD3100 was encapsulated within the liposome and coated on its surface. Based on the results of the Liposomal-AMD3100 study, AMD3100 was more effective in remodeling the immune and stromal microenvironment than AMD3100 free, suggesting that the liposomal formulation had an improved pharmacodynamic profile. A murine TNBC model (4T1) demonstrated increased antitumor effects and longer survival times when anti-PD-L1 was combined with Liposomal-AMD3100 (Lu et al., 2021). Accordingly, ICB therapy can be applied to previously ICB-insensitive cancer types by delivering CXCR4 inhibitors liposomal to activate the immune system. It has been revealed that CXCR4 is overexpressed and functional in CRC, which has prompted researchers to examine whether it can enhance standard CRC therapy (Xu et al., 2018). In a CRC HCT116 xenograft model, a study assessed the efficacy of a novel peptide antagonist of CXCR4, Peptide R (Pep R) (D’Alterio et al., 2020). Pep R was administered to mice bearing xenografts of HCT116 with chemotherapeutic agents 5-Fluorouracil (5FU) and oxaliplatin, or 5FU combined with radiotherapy (RT-CT). Compared to chemotherapy alone or Pep R alone, which resulted in 2- and 1.6-fold reductions of the relative tumor volume (RTV) after 2 weeks, the combination of chemotherapy and Pep R significantly reduced the RTV fourfold. According to *in vitro* experiments, Pep R inhibited HCT116 cell growth and further reduced the ability of those cells to clone. It was also explored whether Pep R could target epithelial-mesenchymal transition (EMT). A decrease in ECAD expression and an increase in ZEB-1 and CD90 expression were observed with chemotherapy treatment. Pep R restored the pre-treatment expression levels. Pep R also reduced a population of CD133^+^CXCR4^+^ cells in HCT116 and HT29 cells, considered stem-resistant cancer cells (D’Alterio et al., 2020). In general, the findings suggest that targeting CXCR4 with Pep R enhances the effectiveness of colon cancer treatment by reducing stem-resistant cancer cell proliferation, reversing EMT-induced markers, and inhibiting cell growth. Clinical studies are needed to explore this further.

In our recent study, we showed the potential significance of N, N″-thiocarbonylbis(N′-(3,4-dimethylphenyl)-2,2,2-trifluoroacetimidamide) (A1) as a potent inhibitor of the CXCR4 chemokine receptor in the context of CRC therapy (Khorramdelazad et al., 2023). Compared with established CXCR4 inhibitors, A1 exhibited notable inhibitory activity, as demonstrated *in silico*. The investigation further revealed that A1 induced a cytotoxic effect on CT26 mouse CRC cells, leading to apoptosis and G2/M cell cycle arrest, in contrast to the limited impact of the control molecule AMD3100. A1’s effectiveness extended to reducing cell proliferation, particularly when combined with CXCL12, and downregulating the expression of CXCR4 receptors in treated cells. The dual-functionality of A1, acting as both a CXCR4 inhibitor and a cytotoxic agent, suggests its potential as a promising candidate for enhancing CRC treatment strategies (Khorramdelazad et al., 2023).

In another investigation, glioblastoma multiforme (GBM), a highly invasive and resistance-to-treatment brain tumor, was addressed. GBM’s resistance and invasiveness are contributed to by aberrant p53 function influenced by overexpressed MDM2 and MDM4 proteins, as well as an increase in CXCR4 expression (Daniele et al., 2021). This study examined whether inhibiting the p53-MDM axis could enhance the sensitivity of GBM cells. A dual MDM2/4 inhibitor, RS3594, and a CXCR4 antagonist, AMD3100, were used to treat human GBM cells and GBM stem-like cells and in addition to inhibiting neurosphere growth and inducing differentiation of GBM cells, AMD3100 and RS3594 demonstrated synergistic effects on cancer stem components (Daniele et al., 2021). It appears that simultaneous blockade of CXCR4 and MDM2/4 may offer potential therapeutic benefits in reducing GBM proliferation and invasiveness.

Among the therapeutic challenges associated with triple-negative breast cancer (TNBC), which lacks molecular targets, this study addressed. TNBC tumor growth and metastasis are implicated in the CXCR4/SDF-1 axis, which may be targeted as a therapeutic target. TNBC cells were investigated for their response to Saikosaponin A (SSA), a compound derived from Radix bupleuri (Wang et al., 2020). In mouse models, SSA significantly reduced TNBC cell proliferation, colonization, migration, and invasion, inhibiting primary tumor growth and reducing lung metastasis. Notably, SSA decreased CXCR4 expression without affecting CXCR7. Consequently, MMP-9 and MMP-2 expression were inhibited, and the Akt/mTOR pathway was inactivated. Accordingly, SSA tends to exert its effects by inhibiting CXCR4 expression, which makes it an attractive candidate therapeutic agent for TNBC patients (Wang et al., 2020).

### 5.2 Autoimmune diseases

Several pathological processes, such as cancer and inflammatory diseases (Bekaddour et al., 2023), are implicated in aberrant CXCR4/CXCL12 signaling (Mousavi, 2020). EPI-X4 was discovered to be an endogenous peptide antagonist and inverse agonist of CXCR4, suggesting that it could be developed as a therapeutic (Harms et al., 2021). A modified EPI-X4 derivative with increased anti-CXCR4 activity, referred to as JM#21, was engineered by researchers using molecular docking analysis and rational drug design. Among other things, JM#21 suppressed human immunodeficiency virus (HIV)-1 infection more effectively than AMD3100, a small molecule CXCR4 antagonist approved for clinical use. JM#21 did not cause toxic effects in zebrafish embryos, demonstrating its safety. A mouse model of atopic dermatitis revealed that it attenuated allergen-induced immune cell infiltration and prevented skin inflammation. As a novel and potent CXCR4 antagonist, EPI-X4 JM#21 is positioned in the text as a first-in-class inhibitor with therapeutic efficacy in treating atopic dermatitis, highlighting its importance (Harms et al., 2021). This study supports the clinical development of CXCR4 antagonists to address various diseases associated with CXCR4, including asthma and atopic dermatitis.

A study aimed at developing a CXCR4 inhibitor suitable for topical use in treating psoriasis to minimize systemic toxicity (Boonsith, 2017). As a topical drug for psoriasis, the researchers developed PAMD, a polycation derived from AMD3100. In addition to its adaptability for chemical modification, PAMD can be combined with other drugs and form nanocarriers. Due to the localized nature of psoriasis as a skin disease, topical delivery of PAMD improved safety and compliance by targeting the disease locally. A challenge was overcoming the skin’s main barrier, the stratum corneum (SC), which necessitated modifying the technique. It was found that the modified, negatively charged PAMD demonstrated low toxicity in HaCaT cells and good retention and penetration in both healthy and psoriatic skin models after adding citraconic anhydride for a negative charge and oleic acid for lipophilicity. As a result of the modified polymer’s characteristics, several factors influenced the penetrant’s success, including its size, charge, and partition coefficients. The topical administration of AMD3100 and subcutaneous injection of PAMD.COO- significantly reduced psoriasis symptoms in an IMQ-induced psoriasis mouse model. It was found that blocking CXCR4/SDF-1 reduced skin inflammation, as demonstrated by lower mRNA levels of pro-inflammatory cytokines *IL-1β*, *IL-6*, and *TNF-α*. CXCR4 antagonistic polymers were shown to have similar therapeutic effects to AMD3100 administration by topical application. The polymer’s anti-psoriatic activity in mice did not appear to be affected by its penetration, indicating its efficacy in localized treatment without compromising therapeutic outcomes (Boonsith, 2017).

Researchers investigated the role of the CXCL12/CXCR4 axis in chronic PsO-like skin inflammation, where elevated levels of the angiogenic chemokine CXCL12 and its receptor CXCR4 had been previously observed. Using two experimental models, they found that the CXCL12/CXCR4 axis upregulates blood vessels and macrophages in inflamed skin (Boonsith, 2017). With AMD3100, skin inflammation, inflammation angiogenesis, and accumulation of inflammatory cells were effectively reduced in both models. Anti-CXCL12 antibodies had similar anti-inflammatory effects. These findings were confirmed *in vitro*, suggesting that the CXCL12/CXCR4 axis plays a crucial role in inflammation and inflammatory angiogenesis. Taking advantage of these molecular mechanisms may provide insights into the mechanisms underlying vascular activation in psoriasis, a chronic inflammatory skin disease (Boonsith, 2017). Another study explores the role of CXCR4, expressed by basal keratinocytes (KCs), in inflamed skin using a mouse model with specific loss of CXCR4 in K14-expressing cells (Takekoshi et al., 2013). Despite no apparent skin defects in these mice, they showed increased ear swelling, greater epidermal thickness, and enhanced parakeratosis in an IL-23-mediated psoriasiform dermatitis model. This suggests that CXCR4 plays a regulatory role in keratinocyte proliferation.

Further experiments *in vitro* demonstrated that CXCL12, a chemokine, blocked IL-22-induced keratinocyte proliferation and worked synergistically with IL-22 to upregulate suppressor of cytokine signaling 3 (SOCS3), a key regulator of signal transducer and activator of transcription 3 (STAT3), indicating that SOCS3 is required for CXCR4-mediated growth inhibition. In human psoriatic skin, both CXCR4 and SOCS3 were upregulated in the junctional region at the border of psoriatic plaques (Takekoshi et al., 2013). These findings indicate that CXCR4 surprisingly hinders keratinocyte proliferation and mitigates the effects of proliferative cytokines, providing insights into its role in skin inflammation, particularly in PsO.

They play a crucial role in migrating leukocytes across the blood-brain barrier (BBB) during the inflammatory response in the CNS (Pachter et al., 2003). CXCL12 is highly expressed by microendothelial cells throughout the CNS, suggesting it may help maintain the BBB. With AMD3100, a specific antagonist of the CXCL12 receptor CXCR4, this hypothesis was tested in experimental autoimmune encephalomyelitis (EAE) (McCandless et al., 2006). A study demonstrates that infiltrating leukocytes migrate more rapidly into the CNS parenchyma when CXCR4 activation is lost. CXCL12 is expressed on the basolateral surface of spinal cord endothelial cells under normal conditions (McCandless et al., 2006; Liu and Dorovini-Zis, 2009). As EAE progresses, this polarity is lost in vessels where mononuclear cells have extensively invaded the parenchyma. EAE worsened by inhibiting CXCR4 activation during disease induction since mononuclear cells infiltrated the white matter, resulting in decreased perivascular cuffs and more inflammation. It appears that CXCL12 serves as an anti-inflammatory factor in EAE, limiting the infiltration of autoreactive effector cells into the parenchyma by localizing CXCR4-expressing mononuclear cells to the perivascular space (McCandless et al., 2006).

A novel mutant chemokine designed to antagonize CXCR3 and CXCR4 was used to investigate the role of these receptors in T lymphocyte activation and migration to the central nervous system (Kohler et al., 2008). *CXCL11(4–79)* antagonist was developed from truncation mutants with the highest affinity for CXCR3. CXCR3 ligands (CXCL9, CXCL10, and CXCL11) strongly inhibited mouse T-cell migration with this antagonist. *CXCL12(P2G2)*, another synthetic receptor antagonist, minimally activated these receptors but inhibited activating T cells’ migration in response to the drug. These synthetic receptor antagonists inhibited EAE by interfering with the action of CXCR3 and CXCR4 in a mouse model of multiple sclerosis called experimental autoimmune encephalomyelitis (EAE). They also reduced CD4^+^ T cell accumulation in the CNS. The results of further investigation indicate that *CXCL12(P2G2)* inhibits the sensitization phase of the immune response, but *CXCL11(4–79)* inhibits the effector phase. In treating CNS autoimmune diseases, the findings suggest that targeting both CXCR4 and CXCR3 simultaneously may be beneficial (Kohler et al., 2008).

CXCL12 is implicated in the pathological development of RA, particularly concerning the abnormal migration of peripheral immune cells in joints (Ding et al., 2023). There is controversy surrounding the impact of low-dose methotrexate (MTX) on CXCL12 signaling responses in RA despite its widespread use. According to clinical data, low-dose MTX treatment was associated with clinically relevant downregulation of the CXCR4 on peripheral T cells. By suppressing CXCR4 expression, low-dose MTX significantly decreased cell migration in *in vitro* experiments with CD3^+^ T cells. A significant increase in genomic hypermethylation was observed across the promoter region of the *CXCR4* gene in CD3^+^ T cells treated with low-dose MTX. A significant improvement in arthritis pathology was demonstrated by low-dose MTX-mediated downregulation of CXCR4. It was also found that conditional disruption of the *cxcr4* gene in peripheral immune cells reduced inflammation in arthritis mice’s joints and lungs. It is noteworthy, however, that genetic modification in these mice did not affect their clinical scores for arthritis. By downregulating CXCR4 expression, low-dose MTX may impair immune cell migration and exert anti-inflammatory effects on RA patients (Ding et al., 2023). These findings indicate the MTX’s potential therapeutic effects for RA by revealing how it influences CXCL12 signaling and immune cell behavior.

The chemokine *CXCL12* gene polymorphism has been linked to T1D in humans. CXCL12 levels are elevated in the bone marrow of non-obese diabetic mice, a model predisposed to T1D (Leng et al., 2008). NOD mice accumulate naive T cells, Tregs, and hematopoietic stem cells (HSCs) in their bone marrow (BM). AMD3100, an antagonist of CXCR4, mobilizes T cells and HSCs from their BM. By simultaneously inhibiting insulitis and preventing diabetes, this treatment is simultaneously effective. AMD3100 can treat or prevent T1D in humans by altering T cell and HSC trafficking, which supports the hypothesis that elevated CXCL12 expression promotes T1D in NOD mice (Leng et al., 2008).

An emphasis was placed on the role of CXCR4 in diabetic neuropathy, a common cause of painful diabetic neuropathy (PDN). Observations of elevated CXCR4 levels in peripheral nerve samples from diabetic patients prompted an investigation of the effects of three agents in a streptozotocin (STZ)-induced PDN model in rodents and a naive rat model activating CXCR4/CXCL12 signals (da Silva Junior et al., 2020). A diabetic neuropathy model was induced in Wistar rats through intraperitoneal injection of STZ, providing a platform for measuring rat hypersensitivity, levels of IL-6, and the concentration of calcium [Ca^2+^]i inside diabetic synaptosomes. The outcomes designated a significant decrease in hypersensitivity in diabetic rats following intrathecal administration of Phα1β or intraperitoneal administration of AMD3100, while ω-conotoxin MVIIA did not show an equivalent effect. In naïve rats with activated CXCR4/CXCL12 axis, CXCL12 administration induced hypersensitivity, which was alleviated by Phα1β or AMD3100 after 2 h of treatment, contrasting with the lack of effect detected with ω-conotoxin MVIIA.

Moreover, the study investigated the modulation of IL-6 levels and calcium influx in spinal cord synaptosomes, revealing a decline in both parameters following treatment with the examined agents. Conclusively, Phα1β, ω-conotoxin MVIIA, and AMD3100 revealed effectiveness in decreasing hypersensitivity in STZ-induced PDN in diabetic rats and naïve rats with activated CXCR4/CXCL12 axis. The results propose potential therapeutic avenues, with Phα1β implicating voltage-dependent calcium channels in its repressing effects on PDN (da Silva Junior et al., 2020).

It is expected to find vascular, glomerular, and tubulointerstitial lesions in a renal biopsy taken from a patient with SLE (Weening et al., 2004). The Bowman’s capsule parietal epithelial cells of proliferative glomerulonephritis become activated, and CD133^+^CD24^+^ progenitor cells invade the glomerular tuft (Rizzo et al., 2013). It has been shown that an injury to podocytes results in dysregulated progenitor cells expressing CXCR4, along with high expression of CXCL12 in podocytes, which uses renal biopsies and rat models. In rat models, similar changes are observed in the expression of angiotensin II (Ang II) type-1 (AT1) receptors, which appear to be associated with parietal epithelial cell proliferation. Angiotensin-converting enzyme inhibitors have been shown to normalize the Ang II/AT1 receptor/CXCR4 pathways and to result in regression of lesions in patients with severe forms of glomerular proliferative disorders (Rizzo et al., 2013).

The migration, proliferation, and survival of B1a lymphocytes in the peritoneal cavity are influenced by CXCL12 in normal mice. In NZB/W mice, where these cells are self-reactive and expand, they exhibit increased sensitivity to CXCL12 (Ding et al., 2023). CXCL12 is produced constitutively in the peritoneal cavity, spleen, and glomeruli of mice with nephritis. CXCL12 is specific to the NZB genetic background and modulated by IL-10. In NZB/W mice, antagonists of CXCL12 or IL-10 are used early in life to prevent autoantibodies, nephritis, and mortality. Beginning anti-CXCL12 monoclonal antibody treatment later in life, autoantibodies are inhibited, kidney-related symptoms are eliminated, and B1a lymphocytes and T lymphocytes are suppressed. In this lupus mouse model, abnormally sensitive PerB1a lymphocytes to CXCL12 and IL-10 contribute to the development of autoimmunity (Ding et al., 2023). These data suggest the potential for preventing or mitigating autoimmune manifestations by targeting the CXCL12/CXCR4 axis.

Ulcerative colitis (UC) and IBD are examined to explore the immunological significance of the CXCL12/CXCR4 chemokine axis (Mikami et al., 2008). A multifaceted approach to the study is taken, starting with assessing CXCR4 expression on peripheral T cells in patients with active UC, which revealed significant increases compared with normal controls. According to this study, CXCL12/CXCR4 interaction is associated with UC pathophysiology, where increased expression correlates positively with disease activity. Using a murine model of dextran sulfate sodium (DSS)-induced colitis, the study further demonstrates that CXCR4 expression is elevated on leukocytes and that CXCL12 expression increases in colonic tissue when colitis is induced. A CXCR4 antagonist effectively reduces colonic inflammation in the DSS colitis model and the IL-10 knockout mouse model, suggesting it may be a promising therapeutic intervention. In mesenteric lymph node cells, the antagonist decreases pro-inflammatory cytokines, tumor necrosis factor, and interferon (IFN) production while preserving IL-10 production (Mikami et al., 2008). These findings illustrate potential therapeutic avenues for treating IBD, particularly UC, by targeting the CXCL12/CXCR4 chemokine axis, offering new avenues for intervention in this complex inflammatory condition.

## 6 What is clobenpropit?

Clobenpropit is a potent imidothiocarbamic ester characterized by isothiourea with S-3-(imidazole-4-yl) propyl and N-4-chlorobenzyl substituents. Functioning as a highly effective histamine H3 antagonist and inverse agonist (pA2 = 9.93), it demonstrates notable activity as a partial agonist at H4 receptors. This compound induces eosinophil shape change with an EC_50_ of 3 nM. Clobenpropit serves as both an H3-receptor antagonist and an H4-receptor agonist. Classified as an imidazole, an imidothiocarbamic ester, and an organochlorine compound, it is a conjugate base of Clobenpropit (2+) (Pubchem, 2024). It is essential to emphasize that Clobenpropit exhibits a distinctly low affinity for histamine H1R and H2R, registering pKis of 5.2 and 5.6, respectively (Esbenshade et al., 2003). Regarding its pharmacological impact, Clobenpropit is a concentration-dependent inhibitor of [3H]-dopamine transport in SH-SY5Y cells. The inhibitory effect is pronounced, with a maximum inhibition of 82.7% ± 2.8% and an IC_50_ value of 490 nM (pIC_50_ 6.31 ± 0.11) (Mena-Avila et al., 2018). Furthermore, Clobenpropit distinguishes itself as a subunit-selective noncompetitive antagonist when interacting with recombinant N-methyl-D-aspartate (NMDA) receptors. Its inhibitory activity is particularly potent against the NR1/NR2B receptor, with an IC_50_ of 1 μM (Mena-Avila et al., 2018). In therapeutic applications, a combination regimen involving.

### 6.1 Histamine, histamine receptors, and clobenpropit

**FIGURE 5:**
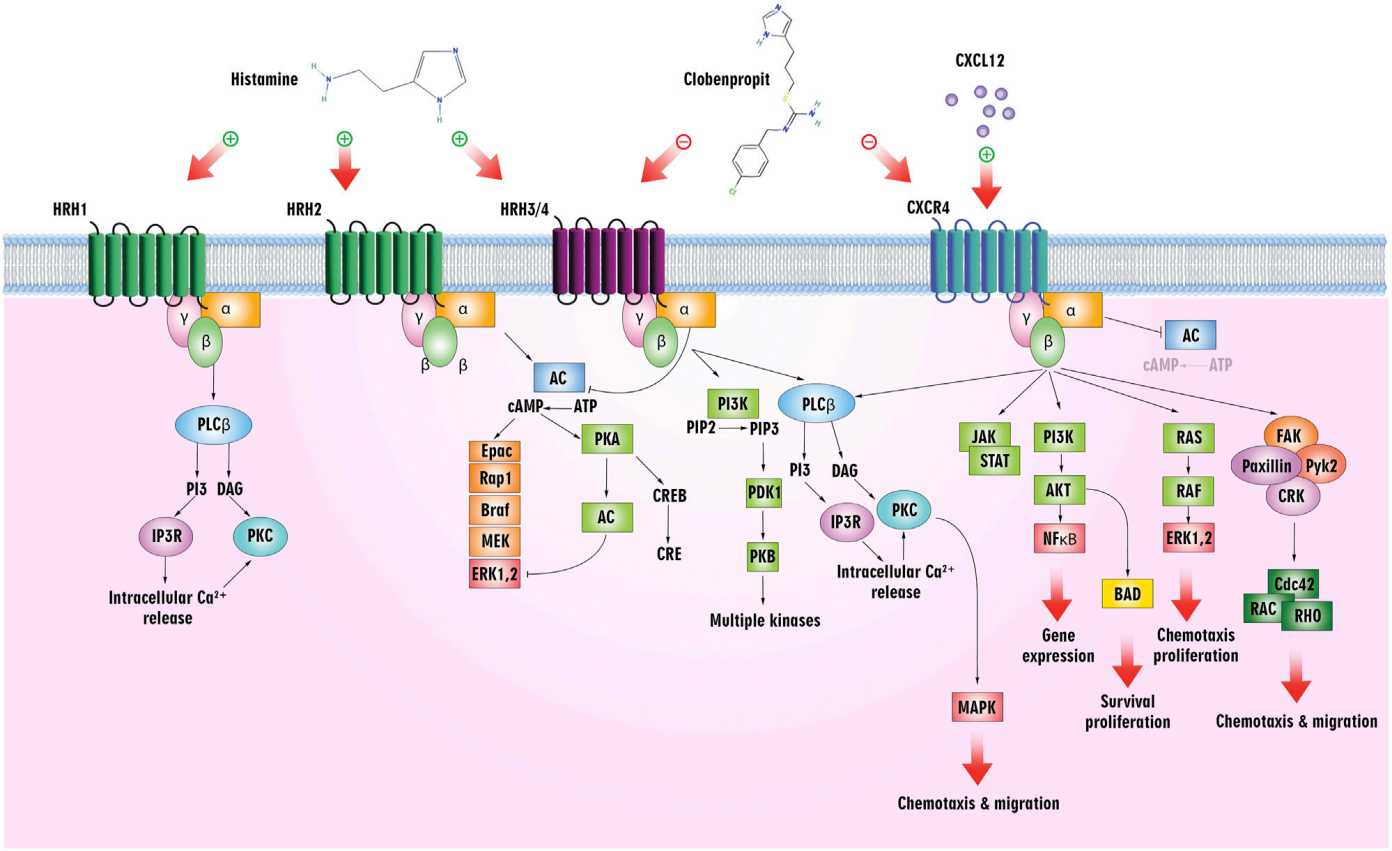
Histamine/histamine receptors and the CXCL12/CXCR4 axis. Clobenpropit binds to H3/H4 and CXCR4 receptors and can inhibit the downstream pathways. Inhibition of these receptors can lead to a decrease in cell proliferation, migration, and production of cytokines, which are used in the treatment of cancer and ADs.

The biogenic amine histamine, synthesized from histidine, has been studied in pharmacology since its discovery in the early 20th century by Sir Henry H. Dale (Dy and Schneider, 2004). Histamine is widely distributed throughout the body and primarily mediates inflammatory processes. The fact that it binds to four GPCR subtypes–H1, H2, H3, and H4 – accounts for its pleiotropic regulatory role in cellular events (Chazot and Tiligada, 2008). In addition to exhibiting differential expressions in different types of cells, histamine shows a broad spectrum of activities. Upon activation, the H3 receptor inhibits cAMP formation, accumulates Ca^2+^, and activates the MAPK pathway. As a target for ligands in treating such conditions, it is implicated in central nervous system disorders (Zampeli and Tiligada, 2009) (Figure 5).

Meanwhile, leukocyte chemotaxis to inflammation sites is mediated by the H4 receptor, mainly expressed in immune cells, including mast cells, monocytes, eosinophils, DCs, T-cells, and NK cells. The H4 receptor more readily absorbs Histamine than the H1 receptor, and activation of the receptor increases intracellular Ca^2+^ concentration (Hofstra et al., 2003) (Figure 5). As an endogenous agonist for the liver-expressed chemokine LEC/CCL16, it contributes to the trafficking of eosinophils (Nakayama et al., 2004).

#### 6.1.1 Histamine receptors in cancer

Various cancer types exhibit heterogeneous outcomes due to the intricate interplay between disparate pathways related to histamine metabolism, the unique landscape of the TME, and the H4 histamine receptor’s central role in signaling cascades (Massari et al., 2020). Histamine has been shown to play a significant role in multiple stages of tumorigenesis, primarily through the H4 receptor, impacting diverse cell types, including cancer cells (Nguyen and Cho, 2021). There is a consistent pattern emerging across a broad spectrum of cancer types, including BCa, CRC, oral tongue squamous cell carcinoma, gastric cancer, melanoma, laryngeal squamous cell carcinoma, bladder urothelial carcinoma, and uterine corpus endometrial carcinoma (Nguyen and Cho, 2021). Compared with normal tissues, tumors exhibit a significant reduction in the expression of the *H4 receptor* gene and/or protein (Nicoud et al., 2019). Additionally, H4 receptor expression correlates with clinicopathological characteristics, suggesting that cancer cell differentiation and tumor progression may be influenced by H4 receptor expression (Fang et al., 2011). The correlation indicates that H4 receptors can be used as a novel prognostic biomarker, providing valuable insights into disease prognosis. It has been reported that partial differentiation in pancreatic cancer is associated with inhibition of cell proliferation through the H1 and H2 receptors (Cricco et al., 2000). Histamine inhibits cell proliferation through the H2 receptor and modulates mitogen-activated protein kinase and Bcl-2 family proteins through the G_0_/G_1_ phase (Martín et al., 2002; Cricco et al., 2004; Cricco et al., 2006). Moreover, a previous study suggests that the H3 and H4 receptors play a role in pancreatic cancer cell proliferation, with the H3 receptor increasing proliferation and the H4 receptor decreasing cell proliferation (Cricco et al., 2008).

Malignancies of the bile ducts, such as cholangiocarcinoma (CCA), are associated with EMT, increasing invasion potential (Vaquero et al., 2017). It has been demonstrated that Clobenpropit, a potent H4HR agonist, inhibits the growth of mammary adenocarcinoma by acting on four receptors (H1-H4) (Patnaik et al., 2018). A study found that cholangiocytes and CCA cells express H1-H4 HRs, and the H3HR inhibits cell proliferation. CCA proliferation, invasion, and EMT phenotypes were significantly reduced by Clobenpropit *in vitro*, affecting the extracellular matrix (ECM). Moreover, Clobenpropit inhibited xenograft tumor growth by disrupting focal contact proteins and altering epithelial and mesenchymal markers *in vivo*. Genetic manipulation confirmed that H4HR was explicitly involved in these effects. Using Clobenpropit to modulate H4HR, CCA cells disrupt their EMT processes, ECM breakdown, and invasion potential (Patnaik et al., 2018).

In another preclinical investigation, for 15 weeks, mice were fed diets containing different test chemicals (terfenadine, cimetidine, or Clobenpropit) to induce colorectal carcinogenesis (Tanaka et al., 2016). Azoxymethane and dextran sodium sulfate (DSS) induced colorectal carcinogenesis in male ICR mice. During week 18, diets containing cimetidine (Hrh2) and Clobenpropit (Hrh3 antagonist/inverse agonist) significantly reduced colonic adenocarcinoma diversity. Colorectal carcinogenesis induced by AOM-DSS was not affected by terfenadine (Hrh1 antagonist). Immunohistochemical analysis revealed varying intensities of adenocarcinoma cells expressing Hrh1, Hrh2, Hrh3, and Hrh4. Inflammation-related colorectal cancer may be accelerated by Hrh2, Hrh3, and Hrh4, according to Clobenpropit, an Hrh3 antagonist and Hrh4 receptor agonist. Additionally, the study provided insight into the molecular aspects of colorectal carcinogenesis that are influenced by histamine receptors by identifying the mRNA expression of pro-inflammatory cytokines and inducible inflammatory enzymes in colonic mucosa (Tanaka et al., 2016) (Table 1).

**TABLE 1:** The most important therapeutic impacts of Clobenpropit in various disorders.

Disease	Mechanism and therapeutic outcomes	Ref
JIA	• Inhibits the production of pro-inflammatory cytokines and chemokines in inflammatory monocytes • Anti-inflammatory effects • Alleviating the hypersecretion of cytokines and chemokines associated with flare-ups	García-Cuesta et al. (2019)
RA	• Reduces cartilage destruction • Reduces bone remodeling • Reduces immune cell tissue infiltration • Reduces pannus formation in animal models of RA • Modulates cytokine production • Anti-inflammatory effect, comparable to the positive response observed with prednisone	García-Cuesta et al. (2019)
AD	• Reduces AβP deposits • Mitigates neuronal and glial reactions • Protective effects against BBB breakdown and edema formation	Gao et al. (2021)
SLE	• Inhibits pDCs and IFN-α production • Downregulating the expression of TRAIL • Inhibits TLR7-activated pDCs by CXCR4	Wang et al. (2010b)
CCA	• Modulates H4 receptors • Disrupts EMT • Reduces invasion potential • Decreases tumor growth	Weir et al. (2011)
Pancreatic cancer	• Combination of Clobenpropit and gemcitabine induces significant apoptosis in tumor cells • Inhibits tumor cell migration • Upregulation of *E-cadherin* • Downregulation of *vimentin* and *MMP-9* • Downregulation of *Zeb1* • Inhibits tumor cell invasion • Inhibits tumor growth	Scharl and Rogler (2012)

#### 6.1.2 Histamine receptors in autoimmune diseases

Various human immune cells express the H4 receptor, which indicates its role in immunomodulation (Nguyen and Cho, 2021). The similar tissue distribution indicates similar physiological roles for this receptor across species despite interspecies differences in amino acid sequence and receptor characteristics. ADs, particularly RA, are associated with histamine. Histamine is considered a pro-inflammatory mediator in arthritic diseases despite its anti-inflammatory properties (Adlesic et al., 2007; Ohki et al., 2007; Grzybowska-Kowalczyk et al., 2008). It indicates that H4 receptors may be related to RA by their expression varying with its severity and duration in synovial cells (Grzybowska-Kowalczyk et al., 2007). Patients with osteoarthritis and RA are found to have H4 receptors within synovial and vascular wall cells, as well as within fibroblasts and macrophages (Grzybowska-Kowalczyk et al., 2008). It has been suggested that the H4 receptor plays a functional role in normal cartilage in rats and that histamine contributes systemically to the arthritic phenotype (Zampeli et al., 2008). This raises fascinating questions about the mechanisms mediated by the H4 receptor in cartilage. According to a comprehensive investigation, Clobenpropit significantly inhibits the production of pro-inflammatory cytokines and chemokines in inflammatory monocytes derived from blood and synovial fluid from individuals with Juvenile Idiopathic Arthritis (JIA) (Bekaddour et al., 2023). A remarkable aspect of Clobenpropit’s anti-inflammatory effects is that it modulates the inflammatory signature observed in patients with JIA rather than targeting specific cytokines. Clobenpropit potentially alleviates the hypersecretion of cytokines and chemokines associated with flare-ups in JIA patients, according to these *ex vivo* findings (Bekaddour et al., 2023). A significant anti-inflammatory effect of Clobenpropit has been demonstrated in animal models of RA, in which IL-6 promotes osteoclast activation, synoviocyte proliferation, and recruitment to inflammatory sites, resulting in synovial pannus development (Lipsky, 2006; Bekaddour et al., 2023). By consistently reducing cartilage destruction, bone remodeling, immune cell tissue infiltration, and pannus formation in collagen-induced arthritis (CIA) mice, the medication clobenpropit significantly reduced cartilage damage, bone remodeling, and immune cell tissue infiltration.

In addition, Clobenpropit diminishes disease progression in arthritic mice and reduces paw thickness, similar to a positive response observed with prednisone as a reference corticosteroid. In a rat model of Alzheimer’s disease (AD) induced by amyloid beta peptide (AβP) infusion, the therapeutic potential of BF 2649 (an H3 receptor inverse agonist) and Clobenpropit was explored. The animals were treated daily for 1 week after 3 weeks of AβP administration. Interestingly, the findings showed that both drugs significantly reduced AβP deposits and mitigated neuronal and glial reactions in the brain. Additionally, a remarkable reduction in BBB breakdown was detected following the treatments and exhibited protective effects against edema formation. Clobenpropit demonstrated superior effects compared to BF 2649. These findings indicate that blocking H3 receptors and stimulating H4 receptors may offer therapeutic benefits in treating AD pathology, providing novel insights into potential treatment strategies (Patnaik et al., 2018).

In Parkinson’s disease (PD), dopaminergic pathways are abnormal, α-synuclein levels are elevated, and tau is phosphorylated in cerebrospinal fluid (CSF). There is a heightened number of histaminergic nerve fibers in specific brain regions of PD, such as the substantia nigra pars Compacta (SNpc), the striatum (STr), and the caudate putamen (CP), as well as an upregulation of H3 receptors and a downregulation of H4 receptors in postmortem cases. By treating SNpC and STr with chronic BF 2649 or Clobenpropit and administering monoclonal antihistamine antibodies (AHmAb), PD-induced brain pathology was reduced significantly. As a result of these findings, revealing the involvement of histamine receptors in PD, novel insights can be gained into developing potential drug strategies for treating the disease, opening up a previously untapped area of research in this field (Sharma et al., 2021).

The immune system’s type I IFNs have vital antiviral and immunomodulatory effects, but prolonged exposure can lead to autoimmune reactions. In SLE, patients exhibit ongoing IFN-α production due to endogenous IFN-α inducers, specifically small immune complexes containing DNA or RNA. These inducers act on naturally IFN-α producing cells, or plasmacytoid dendritic cells (pDC). pDCs are central to innate and adaptive immunity but, through IFN-α production, may contribute to autoimmunity (Rönnblom et al., 2003). It was detected that the histamine receptor 4 (H4R) played a crucial role in mediating the inhibitory impact of histamine on human pDC (Gschwandtner et al., 2011). In this regard, a study explored the effects of Clobenpropit on this process, and the findings showed a more potent inhibitory effect compared to histamine, resulting in a significant reduction of approximately 90% in the levels of IFN-α secreted and membrane TNF-related apoptosis-inducing ligand (TRAIL) expression following HIV-1 stimulation (Smith et al., 2017). Notably, Clobenpropit exhibited no cytotoxic effects at a concentration of 10 μM. This inhibitory effect of Clobenpropit was comparable to that observed with A151, a TLR7 antagonist. Accordingly, it is possible to use this inhibitory impact of Clobenpropit on pDCs and IFN-α production in treating patients with SLE.

An investigation showed that CD14^++^CD16^−^ and CD14^+^CD16^+^ monocyte subsets are activated in systemic juvenile idiopathic arthritis (Macaubas et al., 2012). Their in-depth analysis of protein expression at the single-cell level also revealed the presence of a mixed M1/M2 phenotype at the individual cell level during the flare phase. Consistent with an M2 phenotype, following exposure to lipopolysaccharides (LPS), monocytes express IL-1β; however, they do not release it in systemic juvenile idiopathic arthritis. Despite the inflammatory nature of active monocytes in systemic juvenile idiopathic arthritis, circulating monocytes exibit remarkable anti-inflammatory features. In addition, the perseverance of some of these phenotypes throughout the clinically inactive disease phase claims that this condition mirrors a compensatory response against hyperinflammation (Macaubas et al., 2012).

It has been found that monocytes express the H4R protein, which IFN-γ upregulates (Damaj et al., 2007). Clobenpropit and 4-methylhistamine are H4R agonists that cause monocytes to mobilize Ca2^+^ (Roßbach et al., 2009). In addition, H4R agonists inhibited CCL2 protein production in transmigration assays because they reduced the recruitment of monocytes in supernatants. CCL2 production was downregulated at mRNA and protein levels. Consequently, Clobenpropit can induce a Ca2^+^ influx in monocytes, inhibiting CCL2 production and reducing monocyte recruitment by activating the H4R on monocytes (Dijkstra et al., 2007). It has also been reported that small molecules can inhibit CXCR4 and reduce the polarization of the M1 to M2 phenotype (Song et al., 2021). Therefore, Clobenpropit can also inhibit this phenotype by switching through CXCR blocking. This dual ligation of Clobenpropit to CXCR4 and H_4_R receptors may be beneficial in regulating mixed M1/M2 monocyte-mediated anti-inflammatory responses by decreasing their recruitment and inhibiting prolonged M2-type polarization in some disorders, such as systemic juvenile idiopathic arthritis (Table 1).

Collectively, these data designate the importance of understanding the intricacies of histamine signaling pathways and receptor-specific functions for immune-related disorders and could provide valuable insights into potential therapeutic interventions targeting these pathways.

### 6.2 Molecular docking

**FIGURE 6:**
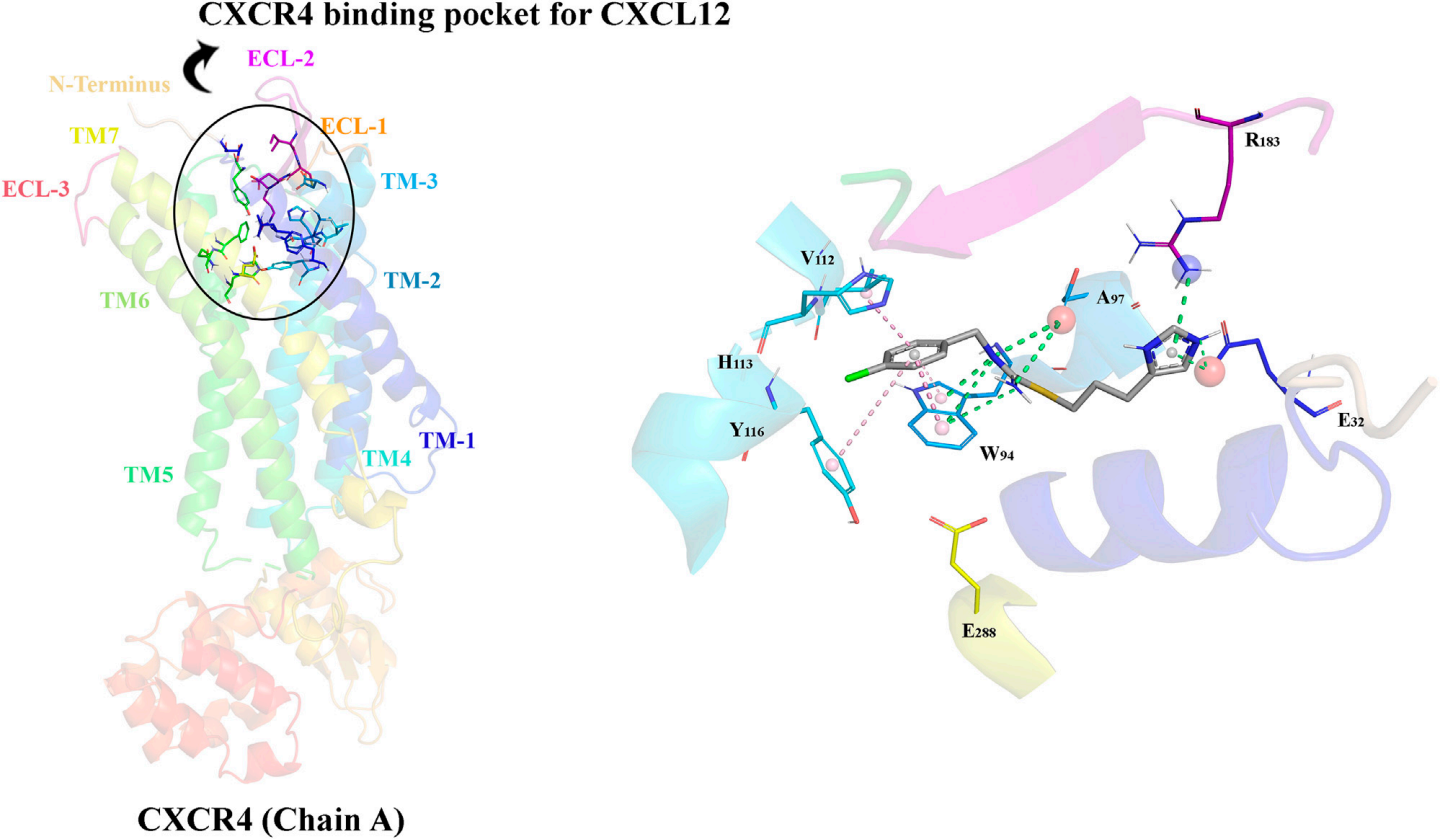
The overall structure of CXCR4 (monomer) and the CXCR4 binding site (groove) for CXCL12 along with a close view of Clobenpropit interactions with CXCR4. Green dashes represent salt bridges and velvet dashes represent π- π stacked and π- π T-shaped electrostatic interactions.

In this study, we explored Clobenpropit interactions in CXCR4 binding pocket for CXCL12 with the aid of molecular docking technique, following our previously performed simulations (Khorramdelazad et al., 2023), and the obtained results were then compared to those of known potent CXCR4 inhibitors, including BPRCX807 (Song et al., 2021), BPRCX714 (Song et al., 2021), AMD3100 (Zhou et al., 2020), WZ811 (Li et al., 2016), MSX-122 (Zhang et al., 2008), as well as ITD, the crystallographic ligand (3ODU) (Table 2). A comparison between the studied ligands’ mode of interactions with that of Clobenpropit shows that the ligand sits very close to those of WZ811 and MSX-122. All three ligands establish π-π stacked and π-π T-shaped electrostatic interactions with residues W94, H113, and Y116. While the hydrophobic pattern is similar in the three ligands, the presence of methanimidamide and imidazole moieties in Clobenpropit structure provides π-cation interactions and salt-bridge formations with E32, W94, D97, and R183 that are not observed in WZ811 and MSX-122 structures and subsequently results in the better binding energy of Clobenpropit for CXCR4 binding pocket for CXCL12. A comparison between AMD3100 and Clobenpropit demonstrates that the presence of positively charged amine groups in AMD3100 structure mediates the formation of salt bridges and charge-charge interactions that enhance AMD3100 binding energy for CXCL12 binding groove significantly (Figure 6). It has already been observed that the creation of salt bridges between ligand and protein acts as clips that stabilize protein structure and can be used as a valuable tool for drug design (Spassov et al., 2023).

**TABLE 2:** Binding energy calculation and the type of proposed compound interactions over CXCR4 binding site for CXCL12. The molecules binding energy values (Except for that of Clobenpropit) are obtained from Nazari et al. (2017).

Compound	MMGBSA (Kcal/mol)	Glide score	Interaction over CXCR4 binding site			
			H-bond	Hydrophobic	Electrostatic	Salt bridge
BPRCX807	−69.67 ± 3.03	−10.32	C186, H203, E288	E32, L41, Y45, D97, W94, A98, V112, H113, Y116, I185, C186, D187, A188, Y190, F199, Q200, H203	W94, H113, Y116	E32, D97, D187, R188
BPRCX714	−64.12 ± 3.03	−8.85	N33, D97, E288	E32, L41, Y45, D97, W94, A98, V112, H113, Y116, I185, C186, D187, A188, Y190, F199, Q200, H203	W94, H113, Y116	E32, D97, D187, E288
AMD3100	−59 ± 3.32	−8.61	—	L41, Y45, D97, W94, A98, V112, H113, Y116, I185, C186, D187, A188, E288	W94, A98	E32, D97, D187, E288
ITD	−47.50 ± 2.00	−7.75	—	L41, Y45, D97, W94, A98, V112, H113, Y116, I185, C186, D187, A188, E288	W94, D97, E288	D97, E288
Clobenpropit	−53.47 ± 0.820	−6.21	—	E32, L41, Y45, W94, D97, A98, H113, Y116, R183, C186	W94, V112, H113, Y116, R183	E32, A98
WZ811	−41.80 ± 0.801	−5.51	Y116, E288	D97, W94, A98, V112, H113, Y116, I185, C186, D187, A188, E288	W94, H113, Y116, C186	E32, D97
MSX-122	−36.71 ± 2.666	−4.76	Y116	D97, W94, A98, V112, H113, Y116, I185, C186, D187	E32, W94, D97, H113, Y116, R183, C186	—

### 6.3 Therapeutic effectiveness of clobenpropit via CXCR4 inhibition

It has been revealed that Clobenpropit inhibits mammary adenocarcinoma spread by acting as a specific H3 antagonist and H4 agonist by decreasing invasion potential (Medina et al., 2008). Several studies have shown that Clobenpropit modulates H4 receptors, disrupts EMT, reduces invasion potential, and decreases tumor growth in CCA (Meng et al., 2011). EMT also plays a vital role in pancreatic cancer metastasis and progression. In addition, Zeb1 is known to make human pancreatic cancer cells resistant to chemotherapy (Arumugam et al., 2009). Therefore, therapeutic agents targeting the EMT process could restore pancreatic cancer’s resistance to chemotherapy.

Combining Clobenpropit (50 μg) and Gemcitabine (5 μg) was reported to be effective (Paik et al., 2014). A study using clobenpropit in combination with Gemcitabine demonstrated that H4 receptors were present and manifested as two subunits, while H3 receptors were not expressed. Clobenpropit inhibited cell migration and increased apoptosis. It has been shown that Clobenpropit induces changes in gene expression associated with cell adhesion and migration, including the upregulation of *E-cadherin*, while the downregulation of *vimentin* and matrix metalloproteinase 9 (*MMP-9*). Compared with Gemcitabine alone, Clobenpropit significantly inhibited tumor growth in a Panc-1 xenograft mouse model, decreasing tumor weight. Correspondingly, Clobenpropit and Gemcitabine synergized to increase apoptosis in the mouse model, owing to the upregulation of *E-cadherin* and the downregulation of *Zeb1*. According to these findings, Clobenpropit, particularly when combined with Gemcitabine, shows efficacy at impeding tumor progression and inducing programmed cell death, suggesting that it may be a promising treatment for pancreatic cancer (Paik et al., 2014).

The CXCR4 receptor is a potential therapeutic target in chronic and acute inflammatory disorders (Smith et al., 2017).

IFNs-I derived from pDCs serve as a cornerstone of antiviral defense and are vital to preventing viral propagation (Khorramdelazad et al., 2022). Dysregulated IFNs-I production, however, can have adverse effects in inflammatory and autoimmune disorders, which is why pDCs must be tightly regulated (Herbeuval and Shearer, 2007). The impact of monoamines and polyamines on pDC function has been uncovered, suggesting that a common receptor mechanism is involved. Researchers have discovered that polyamine derivatives such as spermine phenylguanide and spermidine phenylguanide inhibit HIV-1 CXCR4-tropic strains, suggesting an intricate interaction with this receptor (Wilkinson et al., 2011). In addition, muroid and human pDCs cannot be developed in the BM without CXCL12/CXCR4 signaling (Kohara et al., 2007; Tassone et al., 2010). In particular, the internalization of CXCR4 plays a pivotal role in attenuating pDC activation, delineating an important immunomodulating mechanism. According to findings in a study, Clobenpropit induces CXCR4 internalization in pDCs, whereas FFN-511 (a fluorescent amine mimicking serotonin) colocalizes strongly with CXCR4 (Smith et al., 2017). Therefore, inhibiting CXCR4 by Clobenpropit could reduce the pathologic effects of dysregulated pDC-derived IFNs-I production in inflammatory and autoimmune diseases.

In TLR7-activated human pDCs, histamine and its analog Clobenpropit inhibited the production of all subtypes of IFN by engaging CXCR4. In the broncho-alveolar wash, Clobenpropit administration through intranasal spray significantly reduced type I and III IFN secretion in mice infected with IAV (Smith et al., 2017). CXCR4, not histamine receptors, exclusively mediated the anti-IFN activity of Clobenpropit. Clobenpropit may inhibit monocyte-driven inflammation, particularly in RA, due to its prominent expression in various immune cells, including monocytes and macrophages.

The anti-inflammatory activity of Clobenpropit on monocytes has been demonstrated by competitive experiments using AMD3100 as a CXCR4 antagonist and siRNA-based approaches for reducing CXCR4 expression (Smith et al., 2017). In addition to emphasizing the role of CXCR4 signaling in IFN pathway regulation, this study indicates CXCR4’s role in regulating inflammation in a broad range of cell types, including monocytes and pDCs. Considering the prevalence of type I IFNs in RA and their functional activity, the concurrent suppression of IFNs and anti-inflammatory effects of CXCR4 present significant clinical advantages (Rönnblom and Eloranta, 2013). Researchers have found that individuals with active RA have higher levels of CXCR4 and its natural ligand, CXCL12, in serum and joint synovial fluids. CXCR4 and CXCL12 expression levels were also higher in the group with active RA compared to the group in remission (Peng et al., 2020). It has been suggested that targeting CXCR4 as a therapeutic strategy for RA patients could hold considerable promise because of the increased accessibility of CXCR4.

Compared to JAK inhibitors, Clobenpropit demonstrates its impact at an earlier stage of the inflammatory process (Bekaddour et al., 2023). Instead of directly targeting cytokine-mediated signaling, Clobenpropit intervenes one step upstream by inhibiting the production of inflammatory cytokines. Compared to JAK inhibitors, this unique approach may offer distinct benefits regarding therapeutic effectiveness. In mouse models of RA with Clobenpropit treatment, the marked reduction in disease progression suggests using Clobenpropit-like molecules to target CXCR4 as a potential therapeutic strategy for arthritic conditions (Bekaddour et al., 2023). Clobenpropit is a small molecule with no side effects in *in vivo* preclinical models, indicating that it could be a possible breakthrough drug for treating RA. Clobenpropit modulates cytokine production to exert a comprehensive anti-inflammatory effect by targeting the widely expressed immune cell receptor CXCR4. Clobenpropit combines these attributes to highlight it as a compelling RA treatment candidate (Bekaddour et al., 2023). Regarding the role of CXCR4 in TLR7-mediated inflammation and promising results following treatment of TLR7-dependent lupus-like model with IT1t (a CXCR4 inhibitor), it is possible that administrating Clobenpropit reduces systemic inflammation by suppressing type I IFNs production by pDCs and anti-dsDNA autoantibodies, preventing glomerulonephritis in SLE (Smith et al., 2019) (Table 1).

Clobenpropit and AMD3100 have different mechanisms of action (Bhatt et al., 2010). AMD3100’s main mechanism is its ability to block CXCR4 and activate CXCR7, whereas Clobenpropit modulates histamine H3 and CXCR4 receptors (Kalatskaya et al., 2009; Liao et al., 2013). Compared with AMD3100, clobenpropit exerts unique effects on target pathways, potentially resulting in synergistic or complementary therapeutic outcomes (Figure 5). However, it is noteworthy that AMD3100 may be limited by side effects and resistance development in specific clinical contexts, even though it has demonstrated efficacy in specific clinical contexts (Sideeffects, 2024). In contrast, preclinical studies suggest that clobenpropit may be more tolerated and sustainable as a therapeutic option based on its promising efficacy and safety profiles (Yang et al., 2002). Consideration should also be given to the specific disease context. According to the severity and stage of the condition, some drugs may be more effective than others. Clobenpropit is expected to provide crucial insights for clinical decision-making through comprehensive preclinical investigations and comparative studies.

On the other hand, while several novel small molecules targeting CXCR4 are in clinical and preclinical development phases, Clobenpropit may stand out for several reasons (Debnath et al., 2013; Lu et al., 2024). Clobenpropit demonstrates superior efficacy compared to early-stage drugs. According to our *in silico* studies, Clobenpropit might exhibit the same or even stronger binding affinity or more potent inhibitory effects on CXCR4-mediated pathways crucial for disease progression compared with novel CXCR4 inhibitors, such as WZ811 and MSX-122. This efficacy could be demonstrated through preclinical studies or even early clinical trials. Moreover, Clobenpropit may possess a more favorable safety profile. This aspect is critical in drug development, especially in autoimmune and cancer diseases where patients may already be compromised in terms of their health status. A better safety profile can translate into reduced adverse effects and improved patient compliance and outcomes (Bekaddour et al., 2021). Clobenpropit might exhibit favorable pharmacokinetic and pharmacodynamic properties, such as better bioavailability, longer half-life, or more predictable metabolism (Ishizuka et al., 2008). These factors contribute to the drug’s effectiveness and suitability for clinical use. As discussed, Clobenpropit may act through novel mechanisms beyond mere CXCR4 antagonism (Bekaddour et al., 2021). This could involve additional pathways or synergistic effects that enhance its therapeutic potential, offering a unique advantage over other CXCR4-targeting agents (McHugh, 2019). Clobenpropit may have advanced further along the clinical development pipeline compared to the early-stage drugs if applicable. This could imply a more comprehensive understanding of its efficacy, safety, and dosing regimens through advanced clinical trials or real-world data.

## 7 Concluding Remarks

Since the CXCL12/CXCR4 axis plays a crucial role in cancer and AD pathogenesis, inhibiting it seems like a promising therapeutic approach. Notably, several inhibitors for CXCR4 have been conceptualized and developed, with AMD3100 standing out as one of the most significant achievements, having been approved by the FDA in 2008 and showing efficacy in the treatment of cancer and AIDS, as well as transplantation (De Clercq, 2019). Our molecular docking analyses have unveiled promising binding scores for Clobenpropit compared to other potent CXCR4 inhibitors. Furthermore, the compound’s dual inhibitory action on H3 and H4 histamine receptors warrants attention, given the pivotal roles of these receptors in fostering cancer and ADs, such as RA. Clobenpropit’s concurrent and synergistic impact on these receptors may yield favorable outcomes in the context of cancer and AD treatment.

Even with the intriguing dual mechanism of action, the current shortage of comprehensive studies hampers a conclusive determination of Clobenpropit’s efficacy in inhibiting CXCR4. Further investigations across preclinical and clinical phases are imperative to elucidate the precise role of Clobenpropit in the treatment of malignancies and ADs through CXCR4 inhibition. These studies would not only contribute to substantiating the therapeutic potential of Clobenpropit but also shed light on its intricate interplay with histamine and CXCR4 receptors, thus advancing our understanding of its therapeutic utility.
